# Supplementary figures and images for: Indian genetic heritage in Southeast Asian populations
Source: PLoS Genet. 2022 Feb 17;18(2):e1010036. doi: 10.1371/journal.pgen.1010036 (PMC8853555; doi:10.1371/journal.pgen.1010036)

A

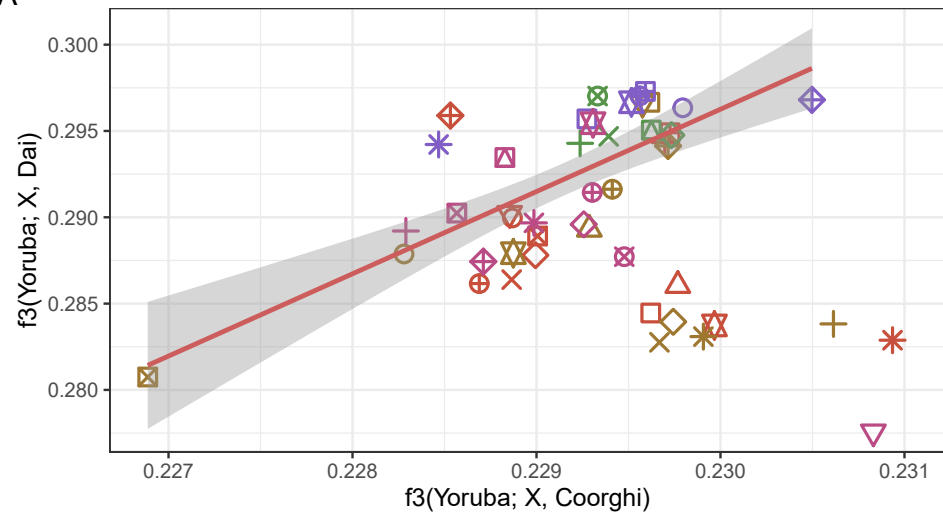

B

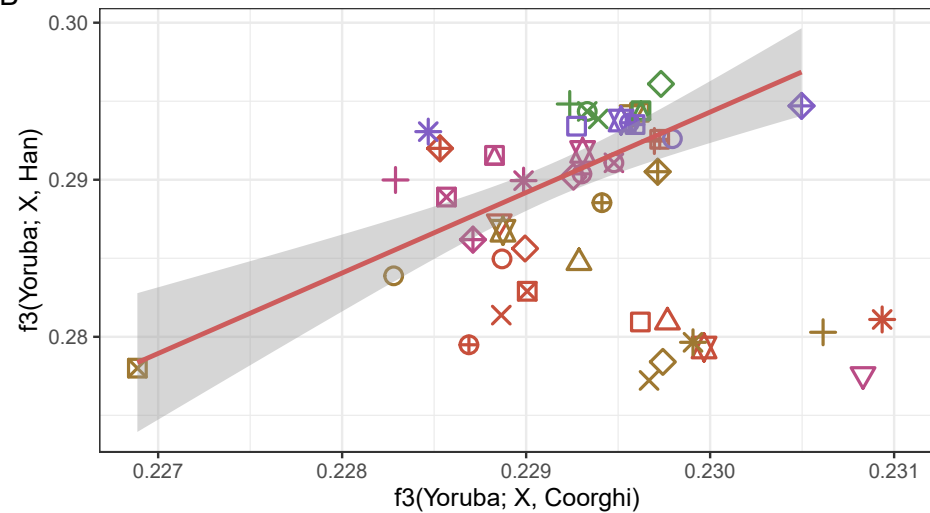

C

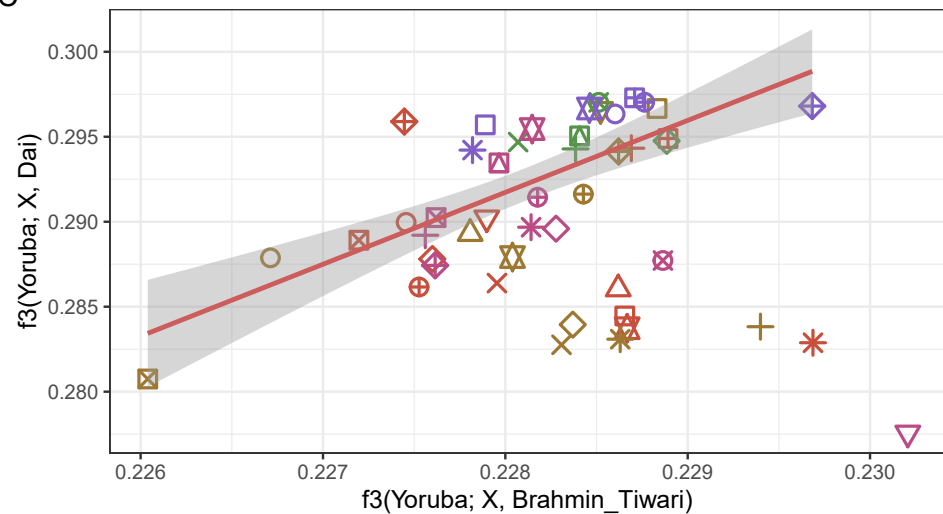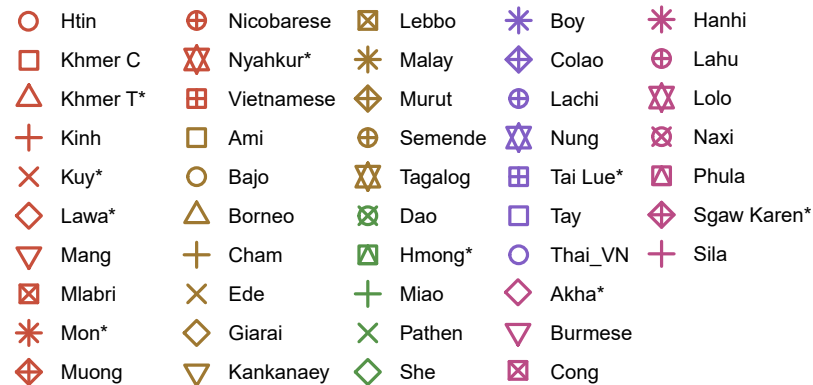

Supplement: S1 Fig — A biplot of f3(Mbuti; Coorghi, X) vs. f3(Mbuti; Dai, X) (A), f3(Mbuti; Coorghi, X) vs. f3(Mbuti; Han, X) (B), and f3(Mbuti; Brahmin Tiwari, X) vs. f3(Mbuti; Dai, X) (C). Asterisks after population names indicate that these populations are newly genotyped in this study. (PDF) [file pgen.1010036.s001.pdf]

**A**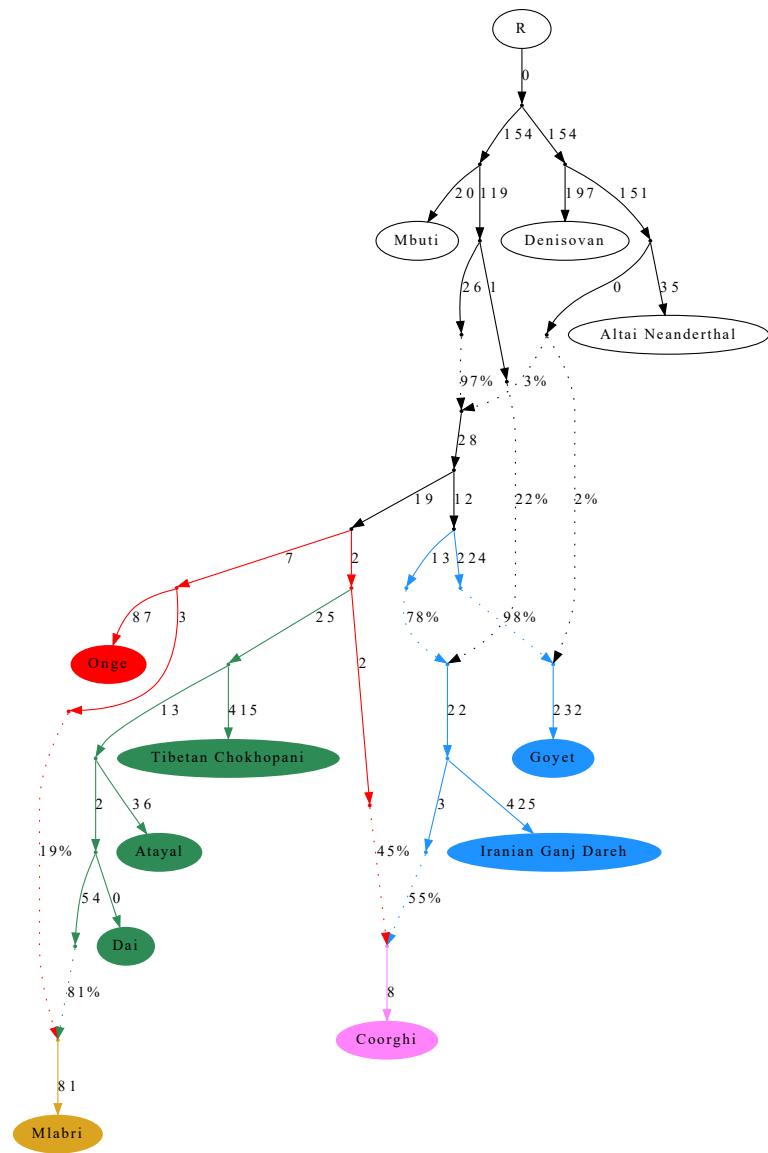**B**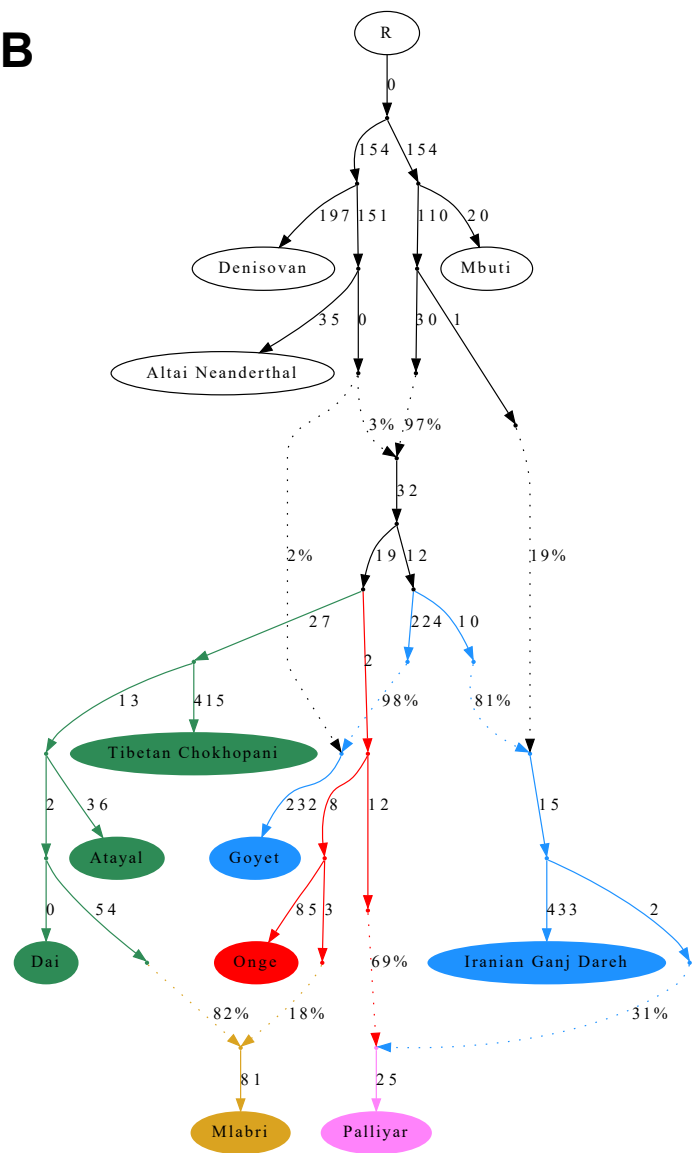

Supplement: S2 Fig — We used the skeleton graphs to explore the genetic makeup of ESEA populations. We used different South Indian populations for two skeleton graphs: Coorghi in panel A and Palliyar in panel B. (PDF) [file pgen.1010036.s002.pdf]

**A**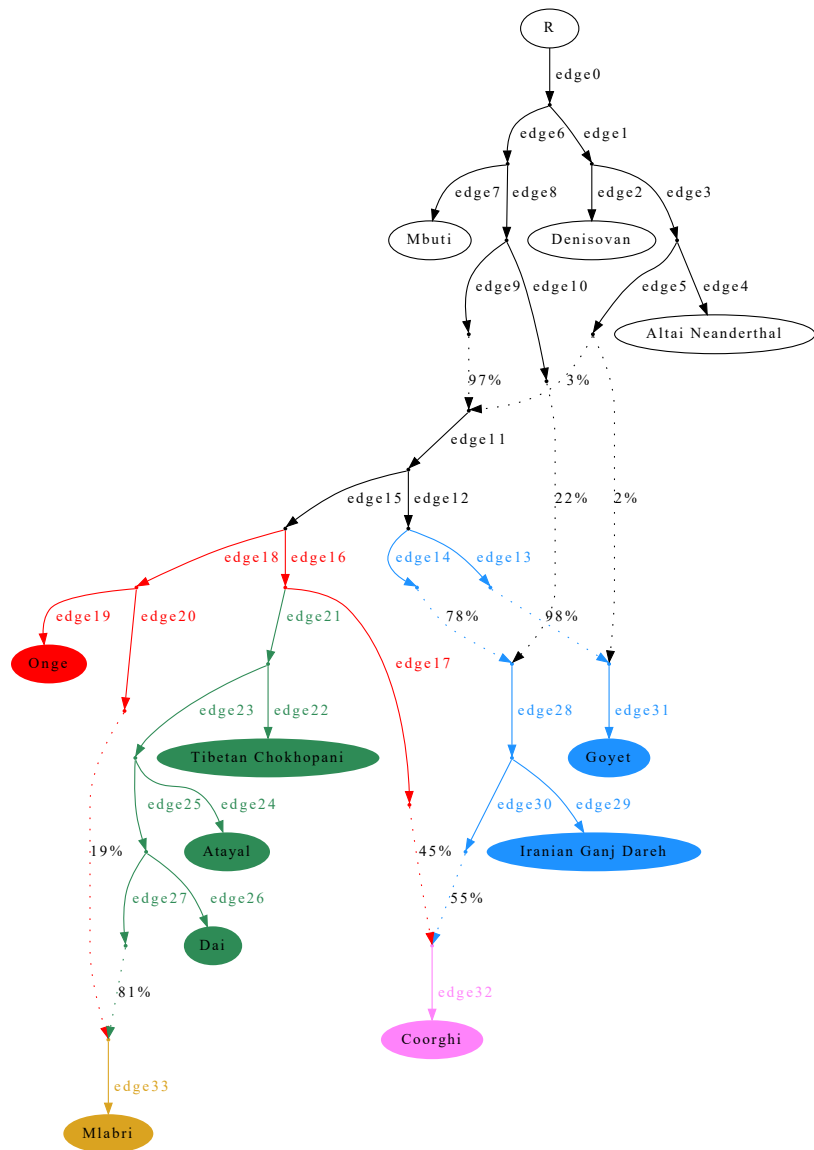**B**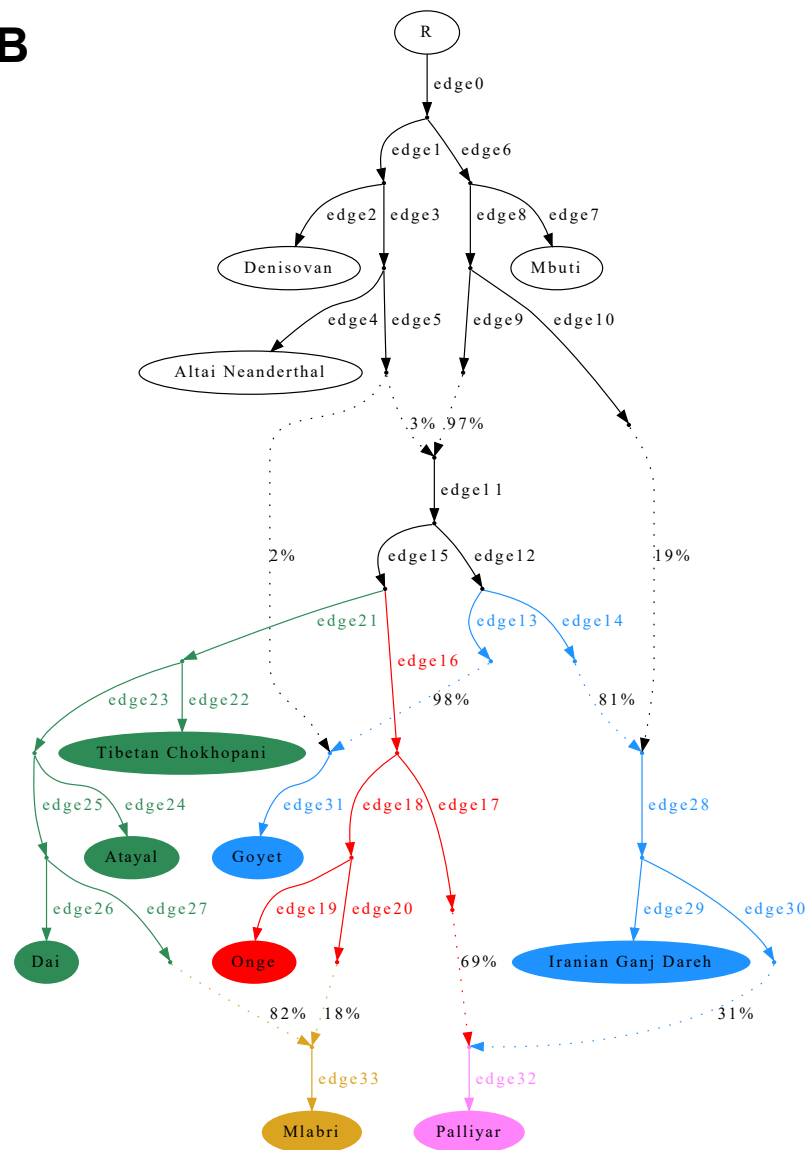

Supplement: S3 Fig — Coorghi was used as an Indian surrogate for skeleton graph (A) and Palliyar for skeleton graph (B). (PDF) [file pgen.1010036.s003.pdf]

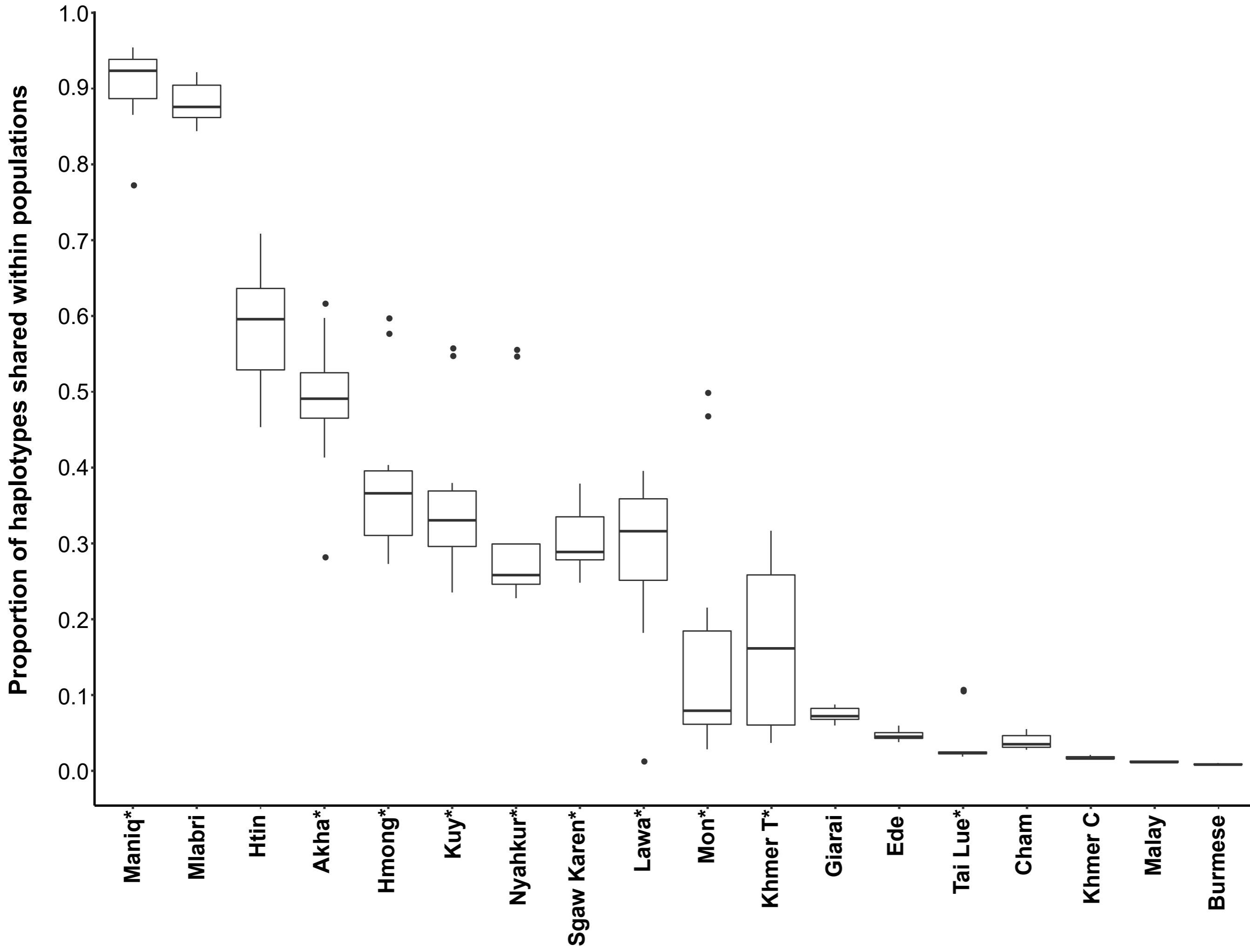

Supplement: S5 Fig — The plot represents proportions of shared haplotypes within-population to total shared haplotypes. Haplotype-sharing was inferred using ChromoPainter v.2. Asterisks after population names indicate that these populations are newly genotyped in this study. (PDF) [file pgen.1010036.s005.pdf]

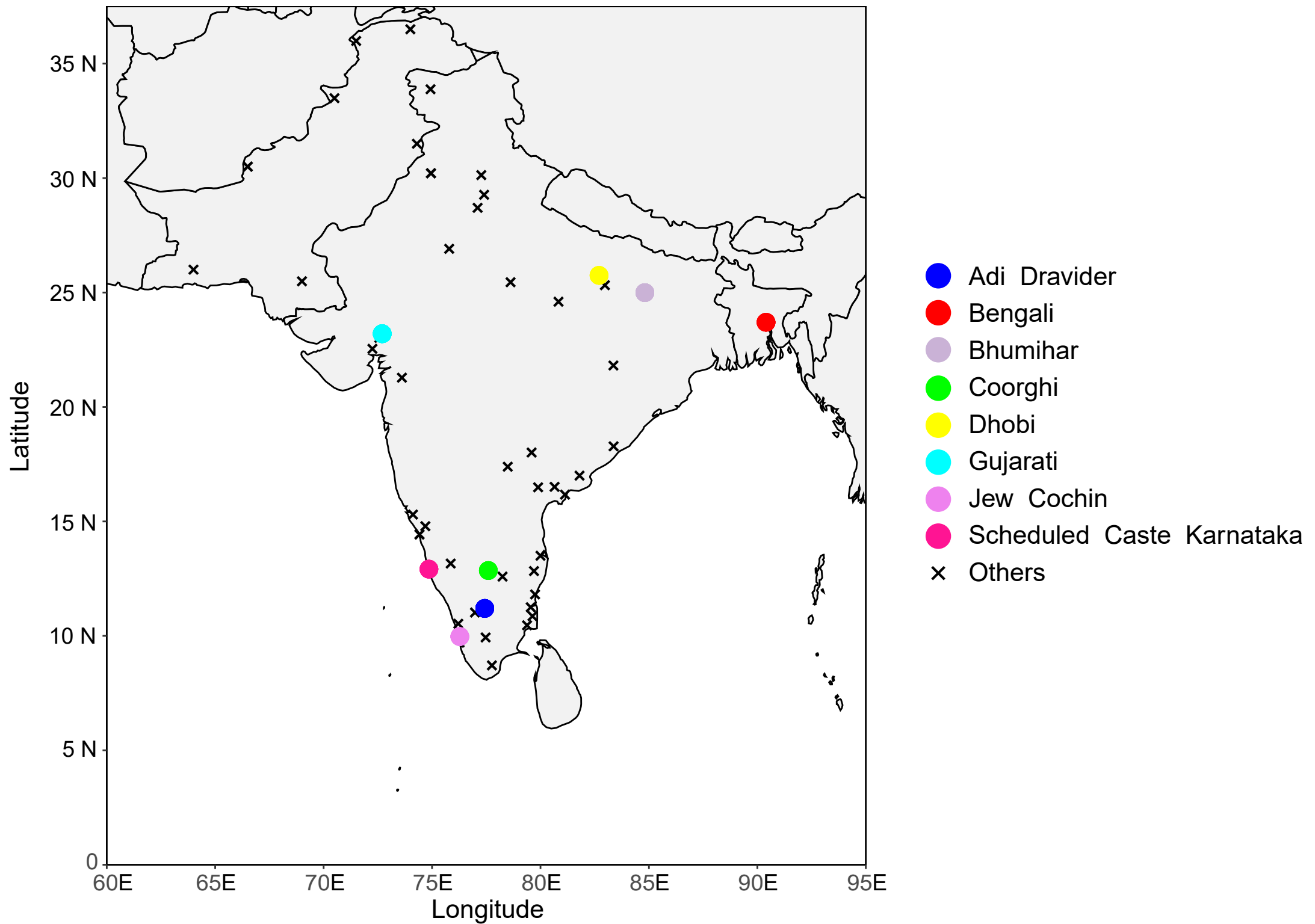

Supplement: S6 Fig — Colored circles represent South Asian surrogates contributing at least 1% of ancestry in any MSEA target group. South Asian surrogates which contribute less than 1% of ancestry are labeled as Others. The map was plotted using an R package “rnaturalearth” (https://github.com/ropensci/rnaturalearth) with Natural Earth map data (https://www.naturalearthdata.com/). (PDF) [file pgen.1010036.s006.pdf]

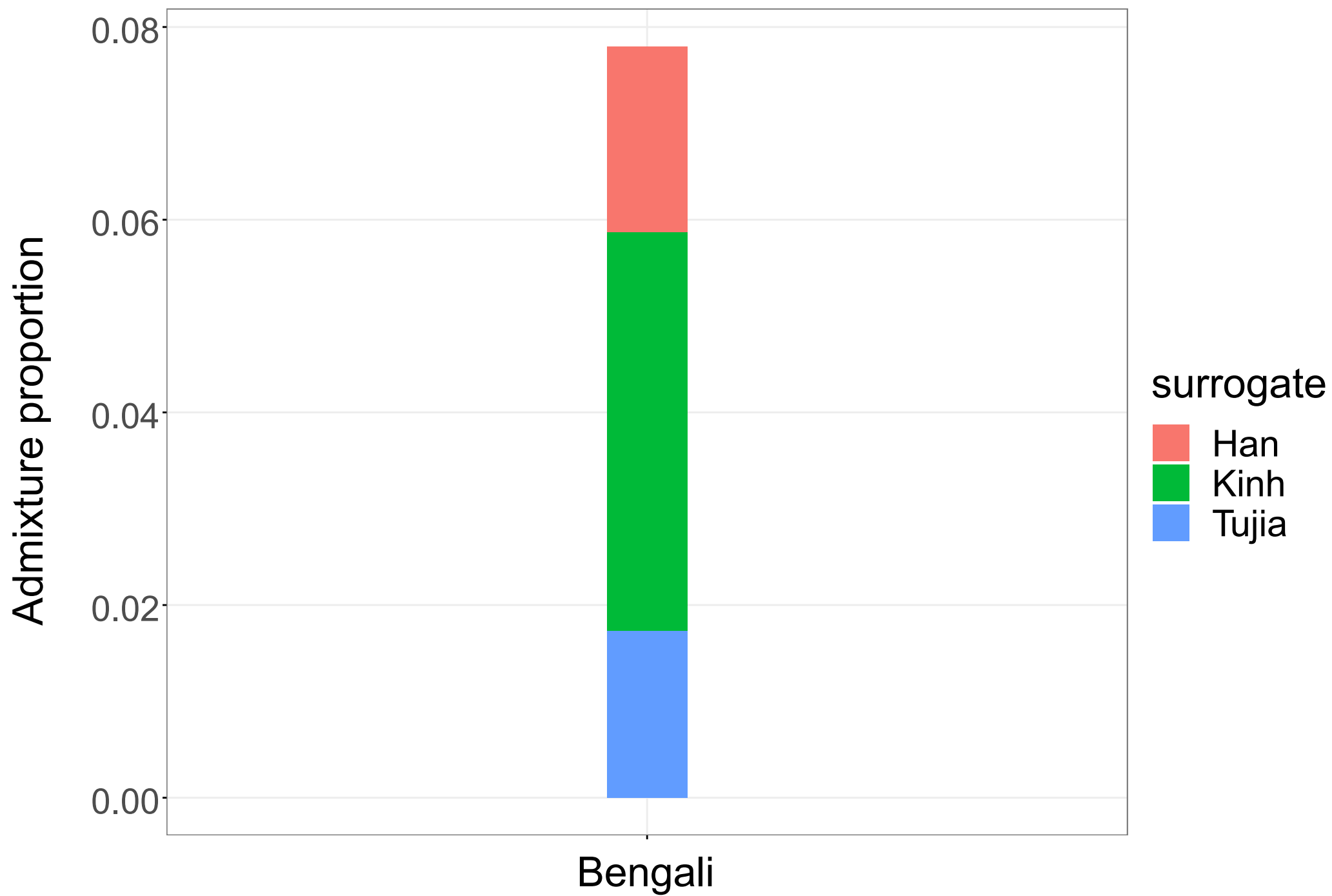

Supplement: S7 Fig — (PDF) [file pgen.1010036.s007.pdf]

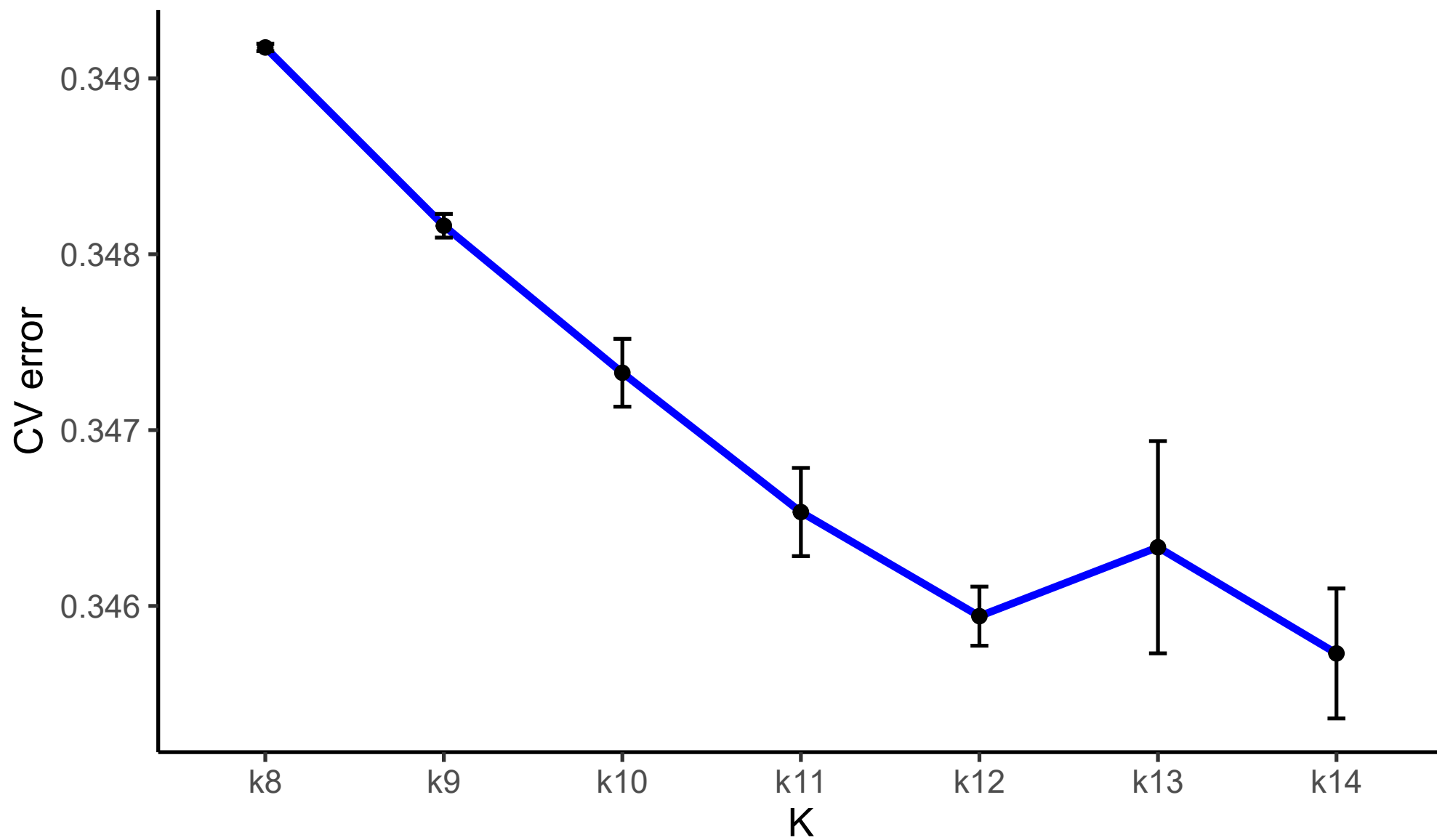

Supplement: S8 Fig — (PDF) [file pgen.1010036.s008.pdf]

template\_first4popNG2\_lock3 :: Alt Den Bel Ata 0.002448 0.000018 -0.002429 0.001139 -2.134

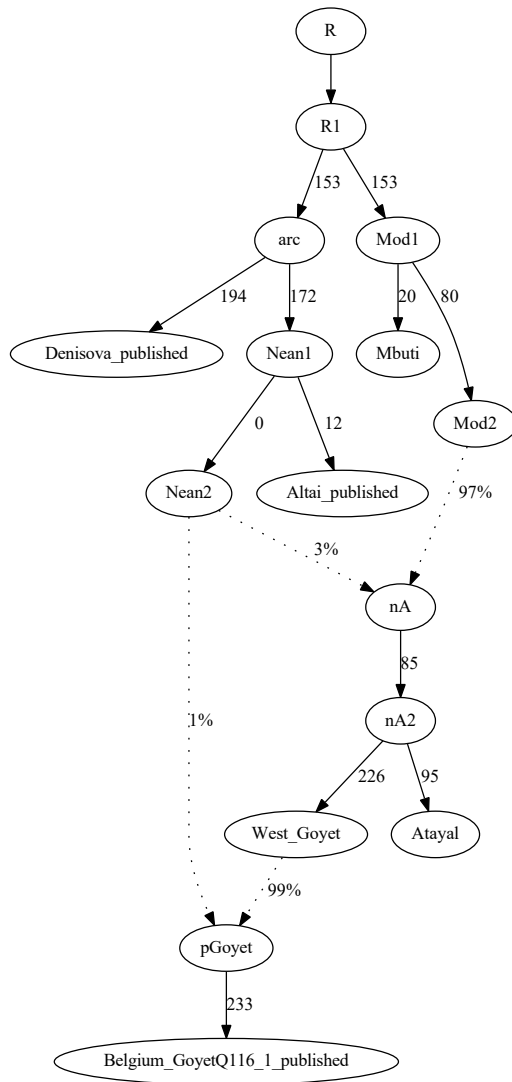

Supplement: S9 Fig — (PDF) [file pgen.1010036.s009.pdf]

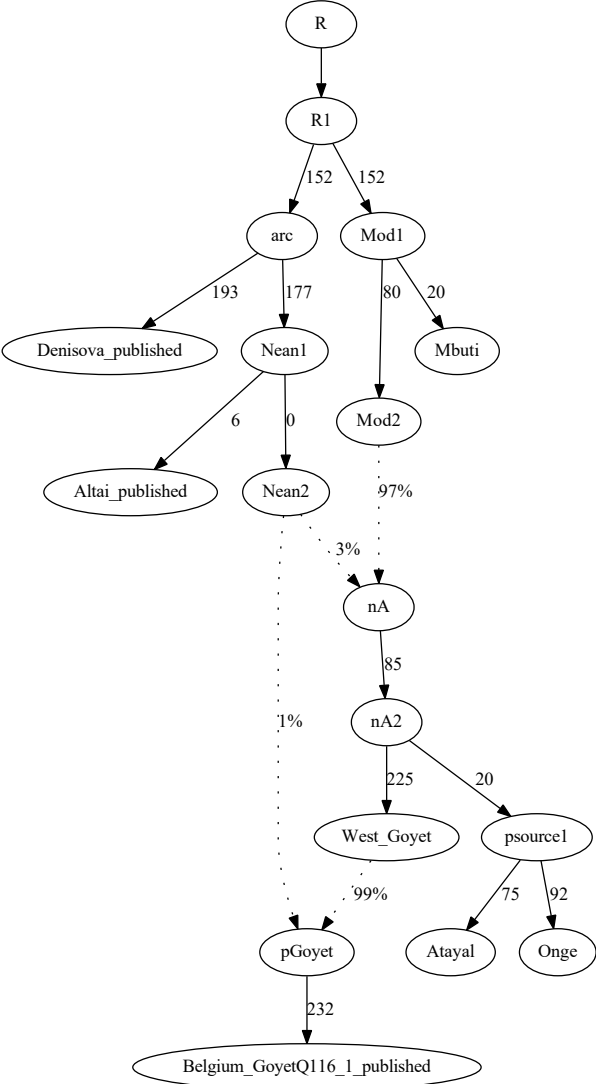

Supplement: S10 Fig — (PDF) [file pgen.1010036.s010.pdf]

template\_first6popNG2\_lock3\_1way\_East\_Atayal\_test\_Dai :: Alt Den Bel Ata 0.002393 0.000018 -0.002374 0.001128 -2.104

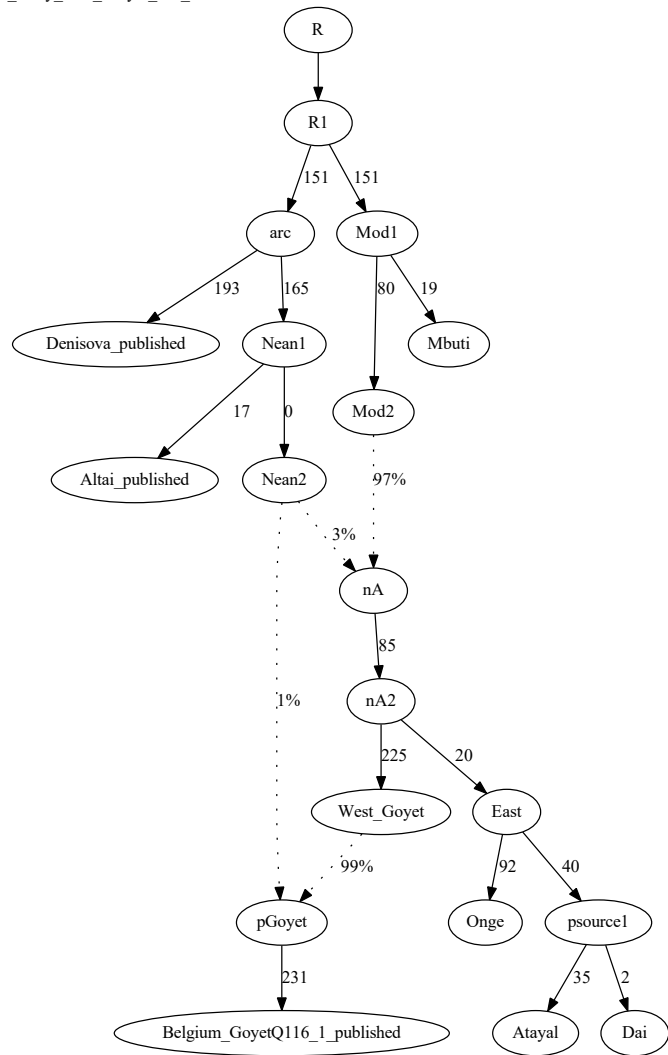

Supplement: S11 Fig — (PDF) [file pgen.1010036.s011.pdf]

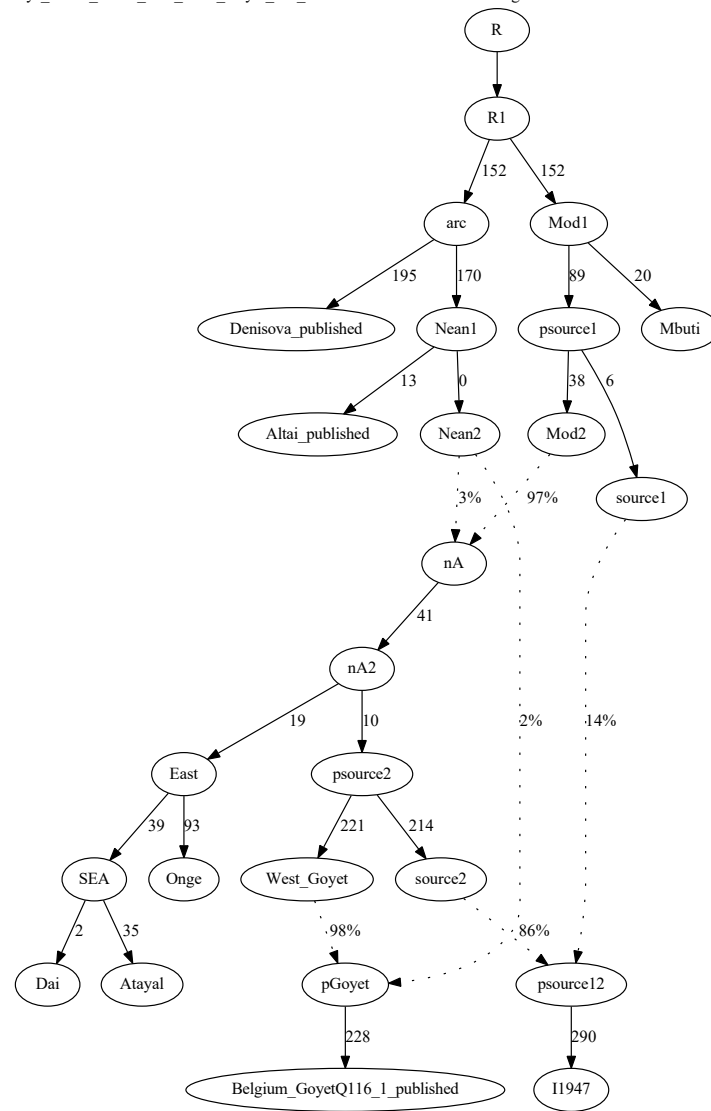

Supplement: S12 Fig — (PDF) [file pgen.1010036.s012.pdf]

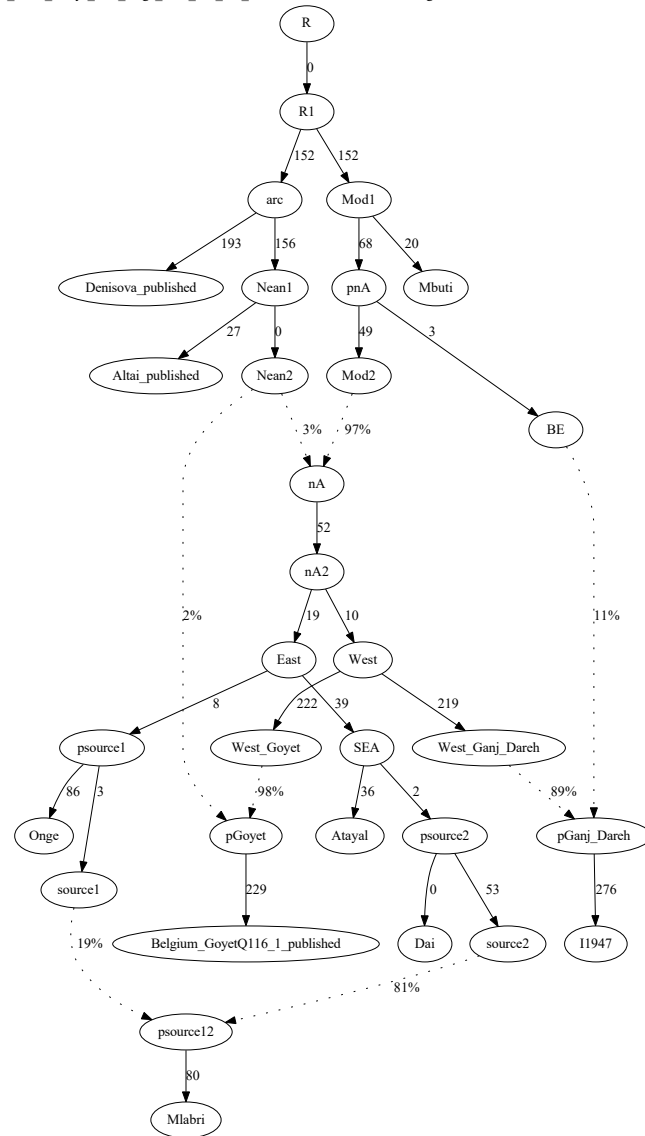

Supplement: S13 Fig — (PDF) [file pgen.1010036.s013.pdf]

**A**

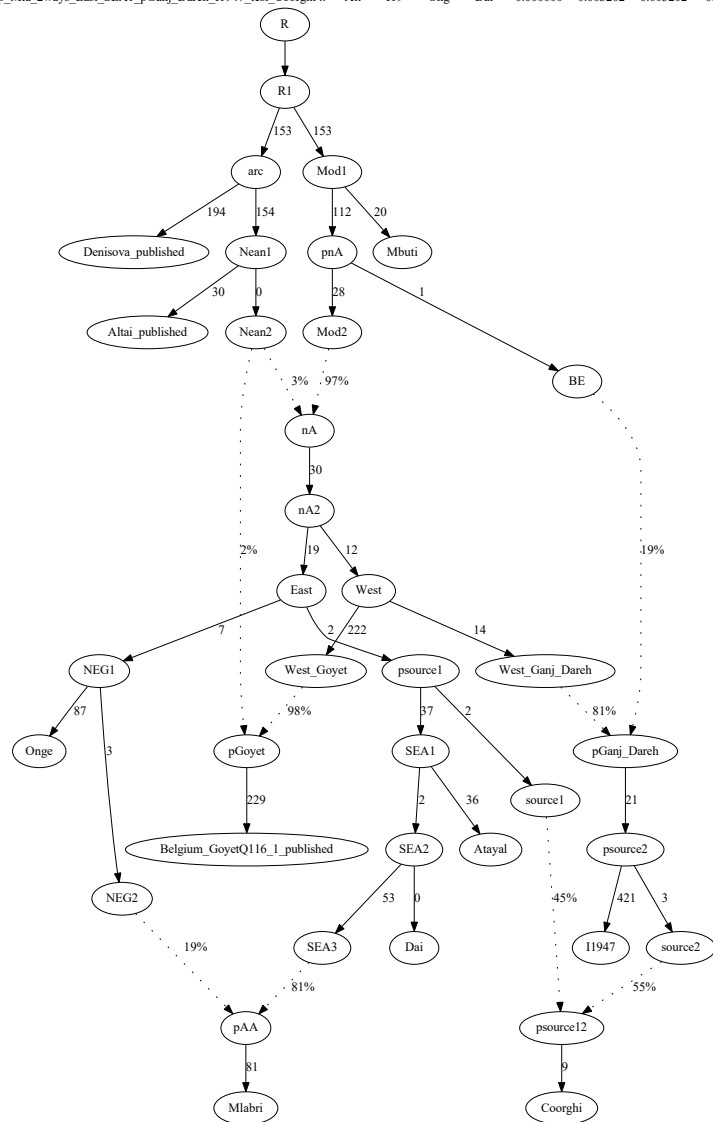

**B**

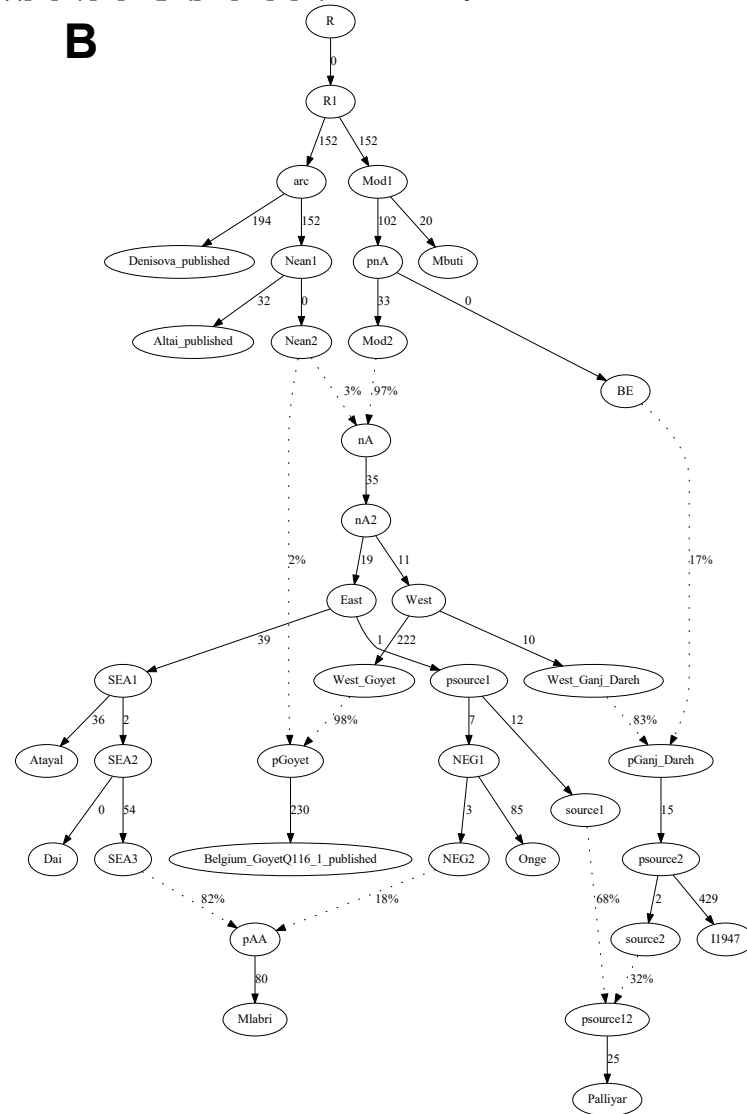

Supplement: S14 Fig — The best-fitting models including Coorghi (A) or Palliyar (B) mapped on the 9-population skeleton graph (S13 Fig). (PDF) [file pgen.1010036.s014.pdf]
