# Supplementary material for: Indian genetic heritage in Southeast Asian populations
Source: PLoS Genet. 2022 Feb 17;18(2):e1010036. doi: 10.1371/journal.pgen.1010036 (PMC8853555; doi:10.1371/journal.pgen.1010036)
Supplement: S4 Fig — Best-fitting qpGraph models for the following Southeast Asian target populations are presented: (A) Akha, (B) Sgaw Karen, (C) Hmong, (D) Htin, (E) Cambodian Khmer, (F) Cham, (G) Ede and Giarai, (H) Malay, (I) Burmese, (J) Tai Lue. Dashed lines represent admixture edges. Each target population was tested separately. qpGraph inferred the same sets of ancestry sources for Ede and Giarai. Therefore, we depict these two populations together in (G). Asterisks after population names indicate that these populations are newly genotyped in this study. (PDF) [file pgen.1010036.s004.pdf]

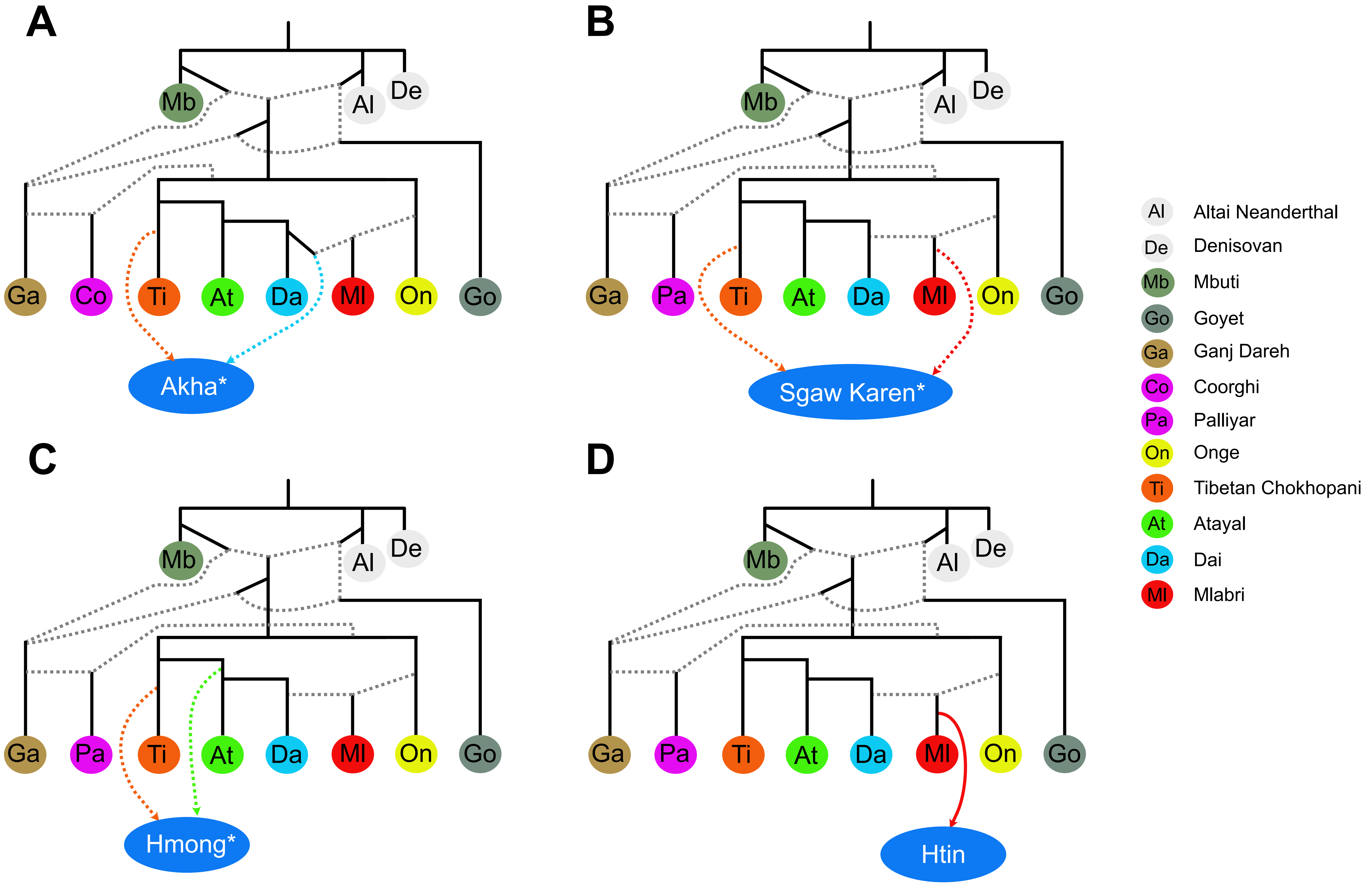

A
B
C
D
Mb
Al
De
Ga
Co
Pa
Ti
At
Da
Ml
On
Go
Akha*
Sgaw Karen*
Hmong*
Htin
Al Altai Neanderthal
De Denisovan
Mb Mbuti
Go Goyet
Ga Ganj Dareh
Co Coorghi
Pa Palliyar
On Onge
Ti Tibetan Chokhopani
At Atayal
Da Dai
Ml Mlabri

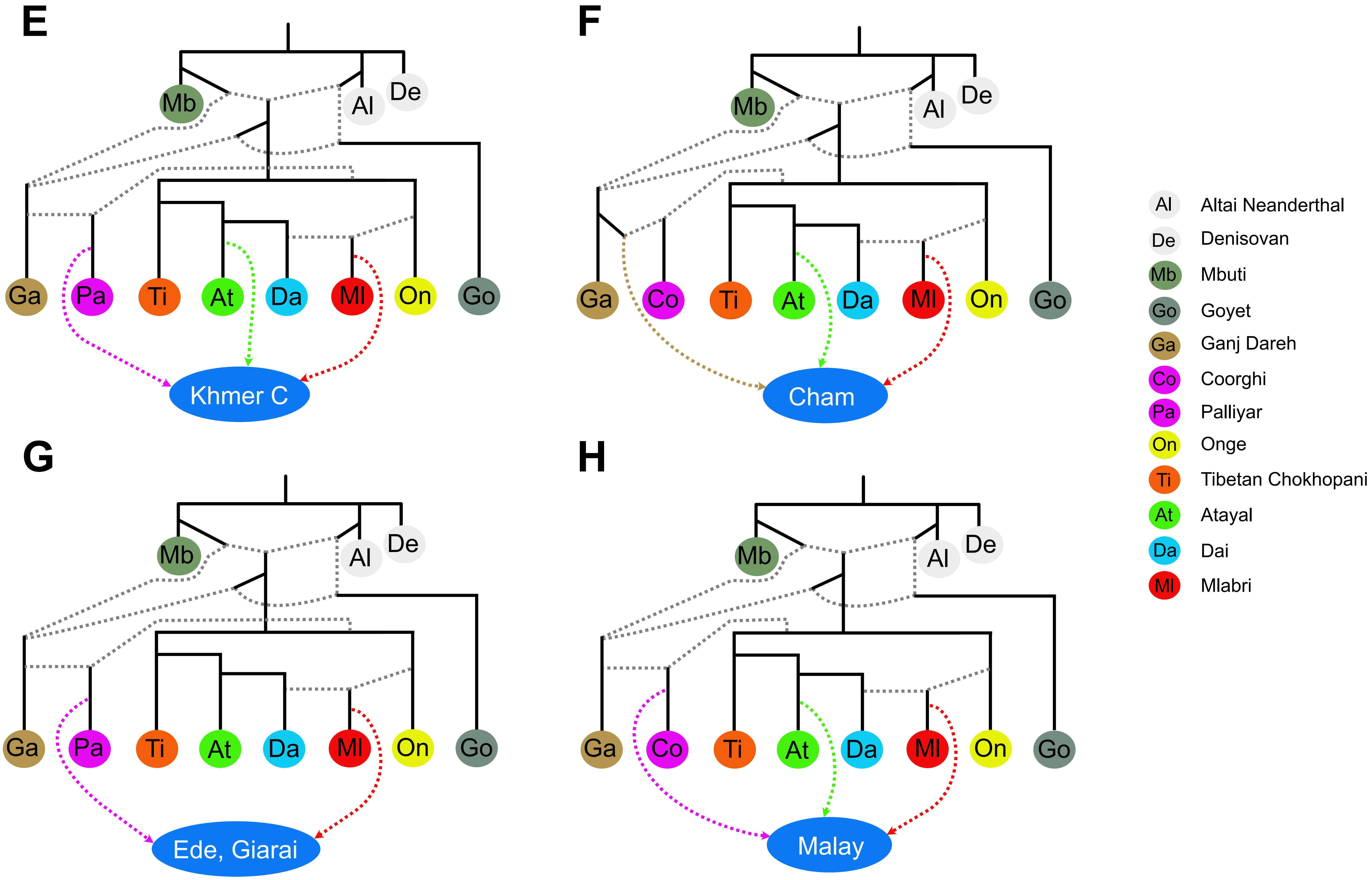
E
Mb
Al
De
Ga
Pa
Ti
At
Da
Ml
On
Go
Khmer C
F
Mb
Al
De
Ga
Co
Ti
At
Da
Ml
On
Go
Cham
G
Mb
Al
De
Ga
Pa
Ti
At
Da
Ml
On
Go
Ede, Giarai
H
Mb
Al
De
Ga
Co
Ti
At
Da
Ml
On
Go
Malay
Al Altai Neanderthal
De Denisovan
Mb Mbuti
Go Goyet
Ga Ganj Dareh
Co Coorghi
Pa Palliyar
On Onge
Ti Tibetan Chokhopani
At Atayal
Da Dai
Ml Mlabri

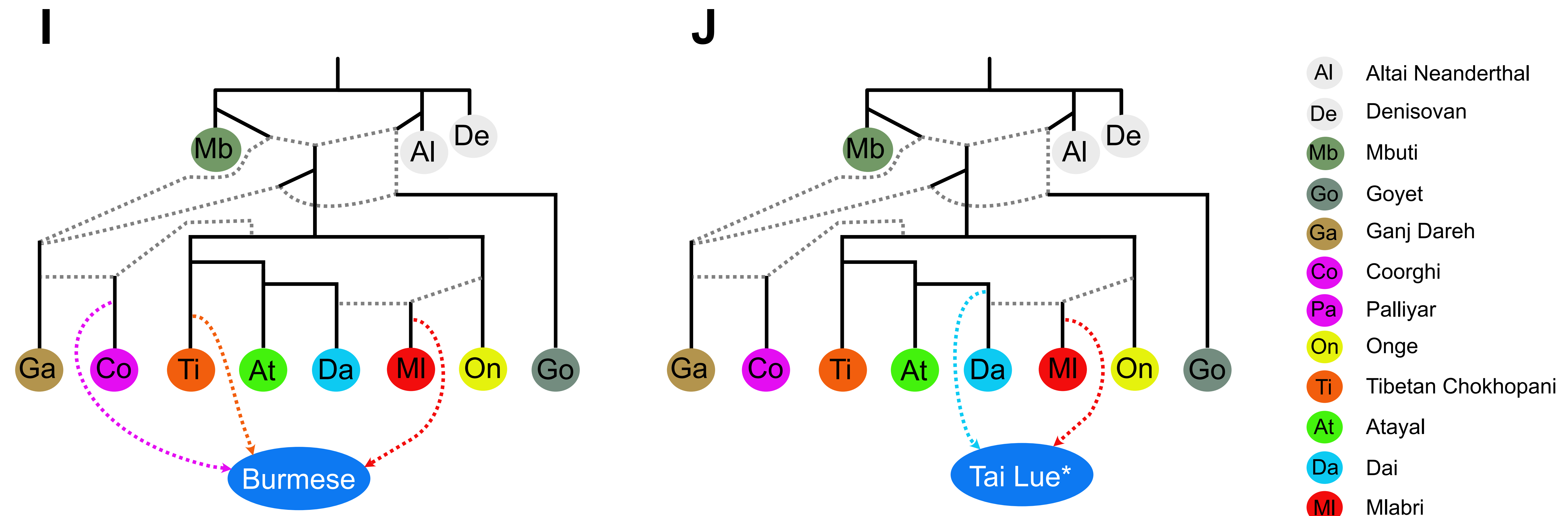
I
J
Mb
Al
De
Ga
Co
Ti
At
Da
Ml
On
Go
Burmese
Tai Lue*
Al Altai Neanderthal
De Denisovan
Mb Mbuti
Go Goyet
Ga Ganj Dareh
Co Coorghi
Pa Palliyar
On Onge
Ti Tibetan Chokhopani
At Atayal
Da Dai
Ml Mlabri
