# Supplementary material for: Indian genetic heritage in Southeast Asian populations
Source: PLoS Genet. 2022 Feb 17;18(2):e1010036. doi: 10.1371/journal.pgen.1010036 (PMC8853555; doi:10.1371/journal.pgen.1010036)
Supplement: Attachment — Submitted filename: response_to_reviewers_final.docx [file pgen.1010036.s019.docx]

Editor: All reviewers think that this manuscript is on a topic of interest within the subject of Human origins and history of major human migrations and that has original results about the role of population movements in spreading cultural practices. However, they have made a number of significant points that should be addressed, as follows:

Reviewer's Responses to Questions

**Comments to the Authors:
Please note here if the review is uploaded as an attachment.**

Reviewer #1: The authors address the potential genetic impact of Indian affiliated states in Mainland Southeast Asia (MSEA). These states emerged in the first millennium CE and had a massive cultural impact on MSEA still plain to see even today. The sample size is not mind boggling but ~120 well-chosen new samples from 10 populations seem to be quite sufficient for the task. The general result is clear. The authors identify Indian genetic ancestry in those MSEA populations who show also Indian cultural ancestry. Importantly, and interestingly, there seems no Indian genetic impact in populations who remained culturally isolated until recently. This is a clear and neat study showcasing an example of genes-culture co-migration.
Perhaps the limitation of the study is that it is a case study and thus difficult to generalize. But this is not to be held against the study as a) it is from many of such studies one would draw general patterns, and b) these results are interesting and relevant in their own right.

The paper would benefit from some reorganisation of the text and some amendments. Introduction already presents the results while it should identify the knowledge gap in the field and the aims of the study. The Results and also most of Discussion is a bit too dry. This latter point is for consideration only. If the authors agree they could try to write it up in a more smoother manner – like the very last paragraph of the Discussion. Figures could be made easier to follow and carry more information.

We thank the reviewer for all suggestions. We implemented many changes suggested and we feel that the revised manuscript is improved substantially. Introduction, Results, and Discussion were modified extensively, as well as the figures. We have reformatted all main text figures according to journal requirements. However, we have noticed that the main text figures in the preview PDF file, generated by the system, have much lower quality than the actual figures. We kindly ask the reviewer to download the main text figures separately for inspection.


Minor comments

Line 37: “We found South Asian low-level admixture in various MSEA populations” better: “We found low-levels of South Asian admixture in various MSEA populations”

We have corrected it as suggested.

Line 82 – 91 This part of the Introduction section strikes as odd. It is indeed a summary of the results. In a way a slightly expanded abstract. Conventionally the introduction section should end with identifying the knowledge gap and the more specific aims of the study in closing (some of) this gap.

We have modified the Introduction by providing a more extensive historical and population genetic background, stating aims of our study, and removing the summary of results from that section.

Figure 1 could be much better. Especially the zoom-in is uninformative in the current state. The background map should show more context. Both geographical, so that one could orientate, and populational – the geographic spread of the populations as opposed to only the sampling locations. The latter is not essential but nice to have.

We have superimposed the distribution of languages on the map and redesigned the figure completely. The inset has been removed.

Figure 2 – Could be better. For the main PCA it would be easier to read if the eight population group names would be spelled out on the figure. If possible indicate also the three main groups of Siberians. The zoom-in is very difficult to follow in the current form. It is easy to lose track in following the symbol shapes on the figure and in the legend. The line width of the symbols in the legend is too thin to actually see the colour. One possibility is using three-letter population codes instead of symbols on the plot itself. And define these in the supplementary table or in the legend. In this way it is easier to follow when one zooms into the plot. Also the colour code for the language groups in the zoom in panel should be shown on the figure.

We used more distinguishable marker colors for the main PCA plot. We also removed several "non-essential" East Asian populations from the plot to decrease clutter in the zoom-in panel. We also increased the line thickness for markers. We tried to spell out population names in the PCA figure as suggested, but in our opinion the modified version presented in the manuscript is more readable than the version with letters shown below.

ADMIXTURE analysis – the choice of K=12 based on highest model likelihood is interesting. In my experience LL values go up as one increases the value of K. There is of course variation so it may be that if one does only 5 replicates then by chance an LL of a run at K=12 is larger than that of runs at K=13. The choice of K is on one hand not perhaps critical as the results at any K are representation of population structure. But some K-s are more easily interpretable in terms of ancestry. What I’m getting at is for example the component maximized in Mlabri is probably there because of very small population size and hence high sharing within the group. In the current representation the figure might suggest a very different ancestry for this population. The same goes for Maniq. Without explanation perhaps a more intuitive K would be better. On the other hand I agree that bringing them out on the figure might make sense. But then there should be more explanation in the text.

Our previous materials and methods section on ADMIXTURE was incorrect. We added a plot of ADMIXTURE cross-validation errors as S7 Fig. We selected K = 12 for presentation as cross-validation errors were not significantly different for Ks from 12 to 14 (S7 Fig), and we chose the simplest of those models. We ranked model likelihoods only among 30 algorithm iterations for K = 12. We subsequently chose an iteration at K=12 with the highest model likelihood. We agree that Mlabri and Maniq are highly drifted groups, as supported by ChromoPainter haplotype sharing statistics (S4 Fig). We mentioned this point in the section describing haplotype-based analyses.

This becomes even more important when one uses a % of a component to group populations. Did Mlabri and Maniq show SAS component at lower Ks? From the following analyses I draw that probably not. But still – using a percent of a component in ADMIXTURE analysis to group populations where the reason some populations may lack the component in question because they are maximized in their own component (due to high within-group sharing), can be misleading.
And why is there white space between Mlabri and Burmese on the figure?

At K = 9 to K = 14, the SAS component is not present in Maniq and Mlabri. We previously left the gap between Mlabri and Burmese in the ADMIXTURE figure to separate MSEA populations with and without the SAS component. Now the gap has been removed.

Line 113: Probably “Figs 3 and 5”. And Fig 3 should show which populations are grouped into Thai 1-3. Does the individual-level variation in SAS component allow grouping populations based on SAS %?

We separated the Thai subgroups according to PCA, ADMIXTURE, and qpWave results, and not only relying on SAS component proportions inferred by ADMIXTURE. We illustrate the differences among the three Thai subgroups in S1 Fig. qpWave cladality tests for pairs of Thai individuals are shown below (and also in S2 Table). Even though qpWave is not able to distinguish Thai2 and Thai3 individuals, qpAdm, qpGraph, and SOURCEFIND results indicate the presence of SAS ancestry in the Thai2 group, while SAS ancestry is missing in the Thai3 individual.

Pairwise qpWave cladality tests for Thai individuals (p-values greater than 0.05, i.e., when cladality was not rejected, are highlighted in green).

|  | pop | Thai1 | Thai1 | Thai1 | Thai1 | Thai1 | Thai1 | Thai1 | Thai2 | Thai2 | Thai3 |
| --- | --- | --- | --- | --- | --- | --- | --- | --- | --- | --- | --- |
| pop | IID | CHI007 | DCH002 | DCH006 | DCH008 | DCH010 | DCH011 | DCH012 | DCH007 | DCH009 | CHI034 |
| Thai1 | CHI007 |  | 0.28784 | 0.08483 | 0.02883 | 0.54961 | 0.38208 | 0.57067 | 0.00362 | 0.13881 | 1.38E-06 |
| Thai1 | DCH002 | 0.28784 |  | 0.50741 | 0.92471 | 0.83121 | 0.58009 | 0.91993 | 9.48E-08 | 0.00021 | 6.68E-13 |
| Thai1 | DCH006 | 0.08483 | 0.50741 |  | 0.33802 | 0.87027 | 0.08175 | 0.58606 | 1.65E-09 | 2.41E-06 | 3.78E-16 |
| Thai1 | DCH008 | 0.02883 | 0.92471 | 0.33802 |  | 0.8229 | 0.35292 | 0.31378 | 4.08E-10 | 6.31E-07 | 1.57E-17 |
| Thai1 | DCH010 | 0.54961 | 0.83121 | 0.87027 | 0.8229 |  | 0.29366 | 0.94102 | 1.83E-06 | 0.00062 | 3.74E-10 |
| Thai1 | DCH011 | 0.38208 | 0.58009 | 0.08175 | 0.35292 | 0.29366 |  | 0.94102 | 5.15E-08 | 0.00051 | 9.09E-12 |
| Thai1 | DCH012 | 0.57067 | 0.91993 | 0.58606 | 0.31378 | 0.94102 | 0.23852 |  | 3.20E-05 | 0.00324 | 2.51E-09 |
| Thai2 | DCH007 | 0.00362 | 9.48E-08 | 1.65E-09 | 4.08E-10 | 1.83E-06 | 5.15E-08 | 3.20E-05 |  | 0.91768 | 0.47404 |
| Thai2 | DCH009 | 0.13881 | 0.00021 | 2.41E-06 | 6.31E-07 | 0.00062 | 0.00051 | 0.00324 | 0.91768 |  | 0.27235 |
| Thai3 | CHI034 | 1.38E-06 | 6.68E-13 | 3.78E-16 | 1.57E-17 | 3.74E-10 | 9.09E-12 | 2.51E-09 | 0.47404 | 0.27235 |  |

Figure 4 (and figure 2 zoom in) The explanation of the colour scheme should be on the figure.

We have added these legends.

Line 122 – I’m a bit confused – on Fig 4 I seem to count more populations shifted toward SAS than are listed in the sentence.

We have now listed these populations (Kuy and Nicobarese) in the text.

From line 128 – This section feels more like Methods rather than Results. Since the qpXXX approaches form the main backbone of the paper – it probably is justified. And it reads well considering it is description of methods. So I’m not recommending doing anything specific, just pointing it out so the authors can decide if they want to change anything.

We agree with the comment, but we decided to keep these methodology descriptions in the text as they could be useful for the readers.

From line 175 – The paper starts to present results based on language affiliation of the populations under study. While this probably makes sense – it comes a little bit out of the blue. This decision should be explained. Better still justified by results obtained so far. For example this setup could be introduced already in Introduction which as it is now ends with summary of results instead.

We have restructured the section, and it is not based on language families anymore.

Overall the results section, especially the qpXXX part could use a softer landing. As it is we go too deep too quickly. I think the readers of PLoS Genetics constitute a group of experts in modern population genetics approaches but a little more context would be helpful. Also for example one of the main results presented in the abstract – the lack of SAS related ancestry in isolated populations – is not easily found/explicitly presented in the main text, except in the last paragraph of Discussion.

We have added explanations and reasons for using the qpXXX analyses, see lines 167 – 177 and 198 - 203 in the revised version. We also emphasized the lack of SAS-related ancestry in relatively isolated populations in the discussion section.

Line 298: This wording “West Eurasian Y haplogroups R1a1a1b2a2a (R-Z2123) and R1a1 were reported in Khmer [29] and Cambodians [30], respectively.” Is a bit too vague. Are these the main Y hgs in these populations? Or is it just that they are present there as minor hgs. I think it would be better to make it more clear. The same construction “were reported in” is used in several places in the Discussion.

Those haplogroups have low frequencies in the populations discussed here. We are now showing Y-haplogroup frequencies in the text.

Line 355 – This section seems not part of the Kra-Dai section. More like conclusion? This paragraph is well written.

That part is indeed a conclusion.

Reviewer #2: Changmai et al. in the manuscript “Indian genetic heritage in Southeast Asian populations” assembled a collection of population genetic samples across mainland Southeast Asia, and tested whether there is pervasive low level of South Asian ancestries in these populations. To this point, the authors did show a low (~2-16%) level of South Asian ancestry across majority of the populations they sampled. However, I feel that despite the stated motivation, the manuscript lacks a narrative around this thesis, and came off as a systematic application of qp-softwares of the populations they assembled. For example, there were descriptions and statements of the qp-analysis results, but no proposal of mechanisms. It seems the authors constantly shifted between a survey of the region with little depth, or a focus on the newly generated data from Thailand. As a result, the study lacked depth in application of population genetic analysis, and also fell short in illuminating knowledge regarding the formation of people around this region of the world.

We appreciate all the comments by the reviewer which helped us to improve our manuscript. We have reformatted all main text figures according to journal requirements. However, we have noticed that the main text figures in the preview PDF file, generated by the system, have much lower quality than the actual figures. We kindly ask the reviewer to download the main text figures separately for inspection.

Major comments:
1. The authors used really only one type of genomic information – the allele frequencies. Though branded as results that were “consistent across various methods used in this study”, they all more or less reflect the same underlying information. Then, there is really not much insight beyond what could be obtained from a naïve ADMIXTURE analysis of the sample. The authors would improve the impact of the paper by utilizing different sources of genomic information, such as LD-based analysis of admixture timing, or haplotype-based investigation of population structure. The genetic data are all based on modern individual; data quality is presumably high and should be amenable to these advanced analytic tools. The authors would also improve the narrative by commenting on how the set of qp-softwares differ from a vanilla ADMIXTURE / PCA analysis. What do they add above and beyond the vanilla analysis? How to decide which set of numbers to believe if ADMIXTURE results differ from qpAdm results?

We have included new haplotype-based analyses (fastGLOBETROTTER and SOURCEFIND) in this revised manuscript. We also tried to explain why we chose each method in the text (see lines 167-177 and 198-203). In short, PCA and ADMIXTURE were not able to detect the deeply diverged East Eurasian hunter-gatherer (Andamanese-related) ancestry in various MSEA populations. The ancestry was found in several MSEA populations in previous studies (Lipson et al., 2018, McColl et al., 2018). We used qpWave and qpAdm to find plausible models of population formation for MSEA groups. We were able to detect the deeply diverged East Eurasian hunter-gatherer and South Asian ancestry in various MSEA populations using qpAdm. We further modeled genetic history of MSEA populations using qpGraph. qpGraph allows us to model demographic history with a higher complexity than qpAdm, i.e., this method can detect unsampled ancestry sources on an admixture graph. We used qpWave and qpAdm to reject models, and we used qpGraph to compare fits of alternative models. Relying on qpGraph, we obtained more insights into population history than using qpAdm: for example, we detected Tibetan- or Atayal-related ancestry in certain MSEA populations.

We are not able to say what method infers admixture proportions more accurately, ADMIXTURE or qpAdm. Only extensive testing on simulated data can answer this question. However, we tend to believe qpAdm results more. As mentioned above, the deeply diverged East Eurasian ancestry is detectable in MSEA populations using qpAdm, but undetectable using ADMIXTURE. Furthermore, we were not able to explore the genetic makeup of highly drifted populations using ADMIXTURE. In this study, ADMIXTURE inferred unique components reaching ~100% in Mlabri and Maniq, but we were able to model the two populations as 2-way mixtures between the East Asian and the deeply diverged East Eurasian sources using qpAdm.

2. In some cases, why not use the available aDNA data? There are places where I thought Hoabinhian sample could be helpful.

Harney et al., 2021, a recent guideline on qpAdm, recommends not to mix ancient and present-day populations in qpAdm analyses. The Hoabinhian samples don’t have high coverage (~ 50% missing rate for Laos_Hoabinhian, ~ 87% missing rate for Malaysian Hoabinhian when overlapped with HumanOrigins sites). And our skeleton admixture graphs already include 5 ancient populations. Adding a population with a high missing rate will substantially decrease the total number of SNPs and compromise the power of qpGraph analysis.

3. I had trouble understanding the significance of Thai1, 2, and 3. They appeared to be arbitrarily grouped based on average SAS ancestry from ADMIXTURE, but then were not really discussed afterwards. What is the significance of these three groups?

We separated the Thai subgroups according to PCA, ADMIXTURE, and qpWave results, and not only relying on SAS component proportions inferred by ADMIXTURE. We illustrate the differences among the three Thai subgroups in S1 Fig. qpWave cladality tests for pairs of Thai individuals are shown below (also in S2 Table). Even though qpWave is not able to distinguish Thai2 and Thai3 individuals, qpAdm, qpGraph, and SOURCEFIND results indicate the presence of SAS ancestry in the Thai2 group, while SAS ancestry is missing in the Thai3 individual. All our analyses indicate genetic differences between Thai1 and Thai2, such as South Asian admixture proportions (16% vs. 5% inferred by qpAdm (S2 Table)), East Asian sources inferred by qpGraph (Table 1, S3 Table), South Asian sources inferred by SOURCEFIND (Fig 7B), admixture dates inferred by fastGLOBETROTTER (Fig 8).

Pairwise qpWave cladality tests for Thai individuals (p-values greater than 0.05, i.e., when cladality was not rejected, are highlighted in green).

|  | pop | Thai1 | Thai1 | Thai1 | Thai1 | Thai1 | Thai1 | Thai1 | Thai2 | Thai2 | Thai3 |
| --- | --- | --- | --- | --- | --- | --- | --- | --- | --- | --- | --- |
| pop | IID | CHI007 | DCH002 | DCH006 | DCH008 | DCH010 | DCH011 | DCH012 | DCH007 | DCH009 | CHI034 |
| Thai1 | CHI007 |  | 0.28784 | 0.08483 | 0.02883 | 0.54961 | 0.38208 | 0.57067 | 0.00362 | 0.13881 | 1.38E-06 |
| Thai1 | DCH002 | 0.28784 |  | 0.50741 | 0.92471 | 0.83121 | 0.58009 | 0.91993 | 9.48E-08 | 0.00021 | 6.68E-13 |
| Thai1 | DCH006 | 0.08483 | 0.50741 |  | 0.33802 | 0.87027 | 0.08175 | 0.58606 | 1.65E-09 | 2.41E-06 | 3.78E-16 |
| Thai1 | DCH008 | 0.02883 | 0.92471 | 0.33802 |  | 0.8229 | 0.35292 | 0.31378 | 4.08E-10 | 6.31E-07 | 1.57E-17 |
| Thai1 | DCH010 | 0.54961 | 0.83121 | 0.87027 | 0.8229 |  | 0.29366 | 0.94102 | 1.83E-06 | 0.00062 | 3.74E-10 |
| Thai1 | DCH011 | 0.38208 | 0.58009 | 0.08175 | 0.35292 | 0.29366 |  | 0.94102 | 5.15E-08 | 0.00051 | 9.09E-12 |
| Thai1 | DCH012 | 0.57067 | 0.91993 | 0.58606 | 0.31378 | 0.94102 | 0.23852 |  | 3.20E-05 | 0.00324 | 2.51E-09 |
| Thai2 | DCH007 | 0.00362 | 9.48E-08 | 1.65E-09 | 4.08E-10 | 1.83E-06 | 5.15E-08 | 3.20E-05 |  | 0.91768 | 0.47404 |
| Thai2 | DCH009 | 0.13881 | 0.00021 | 2.41E-06 | 6.31E-07 | 0.00062 | 0.00051 | 0.00324 | 0.91768 |  | 0.27235 |
| Thai3 | CHI034 | 1.38E-06 | 6.68E-13 | 3.78E-16 | 1.57E-17 | 3.74E-10 | 9.09E-12 | 2.51E-09 | 0.47404 | 0.27235 |  |

4. Throughout the paper, it was difficult to tell the contribution from the newly generated data. I think this will be clearer if the author focused on their newly generated data, and then clearly indicate when a finding based on newly generated data is supported/replicated from previously generated data from a nearby region.

We tried to get a comprehensive picture of the whole region, thus we selected relevant published data to analyze along with our newly genotyped data. We feel that all additional published data we used are important to fill knowledge gaps and provide insights into population history of our new samples, and we cannot separate them easily. Here are some explanations of this decision.

1) Various published data from MSEA we selected in this study harbor SAS admixture. We report the presence of SAS ancestry in four published MSEA populations (Cham, Ede, Giarai, and Cambodian) that was overlooked in previous studies. In contrast, there are three populations with published data (Burmese, Malay, Thai) for whom SAS admixture was reported before. Kutanan et al. (2020 preprint) reported SAS ancestry in Thai (genetic data from Lazaridis et al., 2014) less than a month before we submitted this manuscript. Our study also shows SAS admixture in four newly genotyped populations. Overall, we report novel detection of SAS ancestry in 8 MSEA populations, which is more than any previous studies on MSEA populations.
2) We have generated new data from two Tibeto-Burman speaking populations (Akha and Sgaw Karen). We would like to test whether Burmese, Tibeto-Burman speakers from a neighboring county in MSEA, would also harbor any ancestry in common with Akha and Sgaw Karen. Mon is also a group with newly generated data. There were historical Mon states in present-day Myanmar. Most of present-day Mon in Thailand are descendants of refugees who migrated from Myanmar in the last few centuries, thus we needed to analyze Burmese along with Mon to test the possibility of genetic interaction between the two groups.
3) Kra-Dai is one of the main language families in MSEA. All our samples are from Thailand, a country with the largest population of Kra-Dai speakers in the region. We would like to explore the link between Kra-Dai-speaking populations in Thailand and other Kra-Dai speakers from MSEA and Southern China.
4) Our qpGraph modeling detected Atayal-related ancestry in most Kra-Dai-speaking populations and in Maniq. We needed to test if we could detect the Atayal-related signal in published Austronesian-speaking populations from MSEA (Cham, Ede, Giarai, and Malay) or in a population such as Cambodians which is known to have historical interactions with Austronesian-speaking groups.

At various places in the Results section, we tried to emphasize more what results rely on newly generated data and what on published data.

5. In the Discussion, some groups had more insight than others (e.g. Austroasiatic and Kra-Dai). However, the discussion was no longer limited to proportion of South Asian ancestry, and seemed to distract from the main thesis as stated. The author may want to re-frame the central question on these specific language groups where they were able to (or were more interested to) derive more insight.

We have restructured the Discussion section and extended it substantially to cover all the questions. We also included updated aims of the study in the Introduction section.

Other comments:
1. Description of Figure 2 in the Results: I think it is worth mentioning how the cline for CAS and for SIB are a little different, more consistent with the geographic expectation (i.e. SIB tending towards more European, CAS more in between Europeans and SAS).

We have added the description (lines 133 – 135): Central Asian and Siberian populations lie between the European (EUR) - SAS cline and the East-Southeast Asian (ESEA) cluster. In agreement with expectations from geography, the Central Asian cline lies between the Siberian and South Asian clines.

2. Please consider changing the color scheme of Figure 2. I have trouble telling Munda from SAS, Onge from SIB, and NEGM from EUR.

We have changed the colors in the figure to make them more distinguishable.

3. Line 107 – how is K=12 chosen? In the Methods cross-validation was mentioned, but there was no explicit statement of how K=12 was chosen for presentation. If it is only be maximum likelihood, then increasing K should always increase likelihood. Are the estimated SAS ancestry consistent across Ks?

Our previous materials and methods section on ADMIXTURE was incorrect. We added a plot of ADMIXTURE cross-validation errors as S7 Fig. We selected K = 12 for presentation as cross-validation errors were not significantly different for Ks from 12 to 14 (S7 Fig), and we chose the simplest of those models. We ranked model likelihoods only among 30 algorithm iterations for K = 12. We subsequently chose an iteration at K=12 with the highest model likelihood. The SAS ancestry proportions across K = 8 to K = 14 are quite consistent (SD across Ks for each population range from 0.5% to 2.6%).

4. Line 111 and Figure 3 – it would be good to indicate which populations are the new data. Also label Thai1/2/3 on the figure as well (although see my comment above as I failed to see the purpose of creating these three subpopulations).

We have indicated newly genotyped populations in the text (line 145) and figure (indicated by asterisks) as suggested. We added a new figure highlighting the PCA and ADMIXTURE results that guided the creation of the Thai subgroups (S1 Fig).

5. Figure 4 labels are actually different from the description in text from line 115-120.

We have corrected it in the text.

6. Why not directly test admixture with f3 statistics? Figure 1 of the PCA appears to show quite a few choices one can pick from to model the two ancestral ancestry.

While direct admixture testing is possible with *f_3_*-statistics, post-admixture drift obscures the signal. Thus, this approach suffers from the same problem as ADMIXTURE. We ran admixture *f_3_*-statistics with a setup "MSEA target = EA + SAS". We used Atayal, Dai, or Lahu as EA and 55 different SAS populations (the same SAS surrogates we used for the qpAdm analysis). In total, we tested 165 pairs of EA and SAS groups per one MSEA target. We did not get any negative value when we used Giarai, Khmer, Kuy, Thai1 and Nyahkur as a target population, but SAS ancestry was found in these groups relying on *f_3_*/*f_3_* scatterplots, qpAdm, qpGraph, and haplotype-based methods. Using scatterplots of outgroup *f_3_*-statistics, we were able to identify more MSEA populations with SAS admixture.

7. It would be good to state the intuition and general goals of qpWave, qpAdm, and qpGraph. Currently the authors only state methodically what was run (line 129-141, which could go into the Methods), without giving an intuition of what these methods are doing and why they are used here.

We have added reasons and explanations of selecting those methods in the text (lines 167 – 177 and 198 – 203).

8. Line 145-148: it is difficult for me to parse the criteria for defining plausible admixture model. For example, “all simpler models should be rejected according to the chosen p-value cut-off” is confusing; shouldn’t the more parsimonious model be preferred? Perhaps the authors should clarify.

There was a mistake in the description of the model plausibility criteria. Here is an updated description of the qpAdm protocol (lines 189 – 196):

We applied two criteria for defining plausible admixture models: a) the model is not rejected according to the chosen p-value cutoff; b) inferred admixture proportions ± 2 standard errors lie between 0 and 1 for all ancestry components. If a model meets these criteria, we consider the model as "fitting" or "passing" (S2 Table), although we caution that the only secure interpretation of qpWave or qpAdm tests is in terms of model rejection, and not model fit [13]. For testing 2-way and 3-way admixture, we constructed models "ESEA + Onge" and "ESEA + Onge + South Asian", respectively (Fig 5, S2 Table).

9. There appears to be no discussion of Figure 5 in the Results. They seem to be quite correlated to ADMIXTURE results (particularly looking at Thai 1/2/3), so what does this analysis actually add over ADMIXTURE?

Fig. 5 just shows admixture proportions averaged across plausible qpAdm models (S2 Table) on a map. The purpose of the figure is to show an overview of qpAdm results. We already cited Figure 5 throughout the text when it is relevant. We also explained the reasons why we chose qpWave and qpAdm modeling in the text (lines 167 – 177). In short, PCA and ADMIXTURE were not capable to detect the deeply diverged East Eurasian (Andamanese-related) ancestry in various MSEA populations. The ancestry has been observed in MSEA populations in previous studies (Lipson et al., 2018, McColl et al., 2018), and we were able to detect the deeply diverged East Eurasian and South Asian ancestry in various MSEA populations using qpAdm.

10. Figure 6 is just the skeleton used for analysis, is it really worth a main figure?

We would prefer to keep the figure in the main text since various positions of ancestry sources on the skeleton are mentioned in the text many times. The skeleton graphs themselves also show the genetic history of the surrogates.

11. Line 237-239: “adding a South Asian-related admixture edge never improved qpGraph model fits significantly when a 3-way model with South Asian admixture was rejected by qpAdm” – I do not understand the significance of this statement. Is this not expected?

We expected that, but it is also possible that the results could be otherwise. For example, qpWave and qpAdm results favor just one stream of ancestry from South China in Tai Lue, but qpGraph results show an additional admixture source from a sister group of Mlabri, who harbor a high proportion of deeply diverged East Eurasian ancestry. The bottom line of this statement in the text is that results from qpAdm and qpGraph are consistent as we did not find a discrepancy in the presence of SAS ancestry in MSEA populations.

12. Table S2 is extremely hard to parse – what is “conclusion”? What are columns N through V on the 3-way qpAdm tab? There was no description of the content of these supplementary tables.

The column "conclusion" indicates whether the model passes our plausibility criteria or not. We have renamed it to “passing the criteria” in the S2 Table. The columns N-Q on the 3-way qpAdm sheet represent a summary of plausible model counts per each target per ESEA source. The columns S-V on the 3-way qpAdm sheet represent the summary of SAS proportions in each target. We have added these descriptions to the table.

13. Line 247 – “high worst residual” should be reworded.

We have removed the phrase.

14. Maybe a quarter of the Abstract seems to be material more suitable for the Introduction.

We have removed the part about Hoabinhian and Neolithic populations from the abstract.

15. Line 37: “low-level South Asian admixture”.

We have rephrased the sentence.

Reviewer #3: The manuscript entitled “Indian genetic heritage in Southeast Asian populations” by Changmai et al. (PGENETICS-D-21-00062) reports the detailed analysis of the genetic structure and admixture history of diverse populations from Thailand, based on SNP array data for 119 individuals. The authors suggest that extensive admixture has occurred between South-East Asian and South Asian populations, possibly because of extensive migrations of South Asians during the “Indianization” of MSEA, as defined by Georges Coedès. The study design and analyses are relevant and straightforward. I appreciate that the authors discuss the accuracy of the methods used (qpGraph in particular), based on another study by the same lab. I nevertheless have a number of major comments.

We are grateful for the suggestions by the reviewer which helped us to strengthen the manuscript. We have reformatted all main text figures according to journal requirements. However, we have noticed that the main text figures in the preview PDF file, generated by the system, have much lower quality than the actual figures. We kindly ask the reviewer to download the main text figures separately for inspection.

- Novelty and robustness of the main conclusion. Evidence for a genetic heritage from South Asians in MSEA populations is not completely novel. It has been suggested by several previous studies, including a recent study based on SNP array data (Mörseburg et al., Eur J Hum Genet 2016 ; Kutanan et al., bioRxiv 2020). This is rightfully acknowledged by the authors; however, because this is the main conclusion of the current study, the authors should extend previous analyses, to strengthen their conclusion. Namely, if the evidence of South Asian gene flow to MSEA is strong, the time and precise geographical origins of this gene flow are not known. I suggest that the authors estimate the time of admixture between South Asian and MSEA populations, using e.g. ALDER. If estimated times are concordant with the historical period for Indian cultural influences in Southeast Asia, this could strengthen the authors’ conclusion. Moreover, I suggest that the authors determine which South Asian population is the best surrogate of South Asian ancestry in MSEA populations, using e.g. SOURCEFIND. There is considerable debate relating to the source of South Asian cultural influences in this region. This should be feasible given that South Asians are relatively diverse, and the authors already have access to 55 different populations from South Asia.

We appreciate this suggestion. We performed additional analyses, SOURCEFIND and fastGLOBETROTTER, to locate potential South Asian ancestry sources and date admixture events. SOURCEFIND found 9 South Asian surrogates which contribute more than 1 percent of ancestry in MSEA populations (Fig 7, S5 Fig). Admixture dates inferred by fastGLOBETROTTER also fall into the periods of ancient Indian-influenced states in MSEA. Now these results are presented in a separate section and discussed extensively.

- The manuscript is lacking historical context. The authors should describe in more details in the introduction the current knowledge about the history of MSEA, and the hypotheses that remain to be tested, relating in particular to the “Indianization” of MSEA (see previous point). The authors could then highlight some of their results that may favor particular hypotheses (the larger Indian admixture proportions in coastal vs. northern Thailand, for example).

We have added more details about historical background in the text (lines 79 – 118). We also stated the aims of our study in the introduction section (lines 118 – 124).

- Discussion about the results for populations speaking different linguistic families is very unbalanced. For Sino-Tibetan and Hmong-Mien speaking groups, there is virtually no discussion of the results, while those for Kra-Dai and Austroasiatic groups are extensively discussed. One option is to focus more the discussion on the main conclusion of the study. Can the authors speculate on the reasons why some groups admixed with South Asians and not others? Is geography the only driver of the patterns observed in Fig. 5? Languages do not seem to have been a barrier against gene flow, as evidence for admixture is found for Sino-Tibetan, Austronesian, Austroasiatic and Krai-Dai speakers.

We have restructured the Results and Discussion sections. They are not structured according to language families anymore. We cite sources suggesting that the MSEA groups harboring South Asian admixture are precisely those populations which were heavily influenced by Indian culture or are hypothesized to be descendants of ancient Indianized states. Those populations most likely interacted with Indians who migrated to MSEA and spread Indian culture to the region. Populations living in relatively isolated areas such as hill tribes and hunter-gatherers probably had minimal contacts with Indian migrants in the past, and indeed they show no evidence of Indian admixture.

- I do not think that the results support a “massive” migration of South Asians, as admixture proportions are ~5-10%. This is not comparable with the massive migrations inferred in Europe (Anatolian farmers, Pontic steppe herders), sub-Saharan Africa (Bantu speakers) or Oceania (Austronesian speakers).

We agree. We have corrected the text

Minor points
- The authors may clarify why some populations were chosen as surrogates for the skeleton graph. Why using (distant) Onge and not Maniq? Why using Coorghi and Palliyar as SAS surrogates?

We used Onge instead of Maniq because we wanted to "map" Maniq on the same skeleton graphs as we did for other populations. Furthermore, Maniq is highly admixed with ESEA populations, and adding Maniq onto the skeleton graph will increase the complexity of the graph. We chose Coorghi and Palliyar because when we tested 55 SAS populations by adding a SAS population on the skeleton graph in S12 Fig, Coorghi and Palliyar belong to a small group of SAS populations which have only one model fitting the data according to our criteria. Furthermore, the skeleton graphs including Coorghi and Palliyar (Fig. 6) have slightly different topologies, i.e., the positions of the deep East Eurasian source for Coorghi and Palliyar are slightly different, but both are among the deepest East Eurasian branches (S13 Fig). We wanted to see if admixture models for target MSEA groups will be consistent on these different skeletons, that was another reason for choosing both Coorghi and Palliyar.

- Please add linguistic groups in the legend of Fig. 1. Please remove the minus sign in “-PC1”.

We have modified the figure as suggested.

- l. 111: clarify here why Thai samples are separated in three groups.

We separated the Thai subgroups according to PCA, ADMIXTURE, and qpWave results, and not only relying on SAS component proportions inferred by ADMIXTURE. We illustrate the differences among the three Thai subgroups in S1 Fig. qpWave cladality tests for pairs of Thai individuals are shown below (also in S2 Table). Even though qpWave is not able to distinguish Thai2 and Thai3 individuals, qpAdm, qpGraph, and SOURCEFIND results indicate the presence of SAS ancestry in the Thai2 group, while SAS ancestry is missing in the Thai3 individual. All our analyses indicate genetic differences between Thai1 and Thai2, such as South Asian admixture proportions (16% vs. 5% inferred by qpAdm (S2 Table)), East Asian ancestry sources inferred by qpGraph (Table 1, S3 Table), South Asian ancestry sources inferred by SOURCEFIND (Fig 7B), and admixture dates inferred by fastGLOBETROTTER (Fig 8).

Pairwise qpWave cladality tests for Thai individuals (p-values greater than 0.05, i.e., when cladality was not rejected, are highlighted in green).

|  | pop | Thai1 | Thai1 | Thai1 | Thai1 | Thai1 | Thai1 | Thai1 | Thai2 | Thai2 | Thai3 |
| --- | --- | --- | --- | --- | --- | --- | --- | --- | --- | --- | --- |
| pop | IID | CHI007 | DCH002 | DCH006 | DCH008 | DCH010 | DCH011 | DCH012 | DCH007 | DCH009 | CHI034 |
| Thai1 | CHI007 |  | 0.28784 | 0.08483 | 0.02883 | 0.54961 | 0.38208 | 0.57067 | 0.00362 | 0.13881 | 1.38E-06 |
| Thai1 | DCH002 | 0.28784 |  | 0.50741 | 0.92471 | 0.83121 | 0.58009 | 0.91993 | 9.48E-08 | 0.00021 | 6.68E-13 |
| Thai1 | DCH006 | 0.08483 | 0.50741 |  | 0.33802 | 0.87027 | 0.08175 | 0.58606 | 1.65E-09 | 2.41E-06 | 3.78E-16 |
| Thai1 | DCH008 | 0.02883 | 0.92471 | 0.33802 |  | 0.8229 | 0.35292 | 0.31378 | 4.08E-10 | 6.31E-07 | 1.57E-17 |
| Thai1 | DCH010 | 0.54961 | 0.83121 | 0.87027 | 0.8229 |  | 0.29366 | 0.94102 | 1.83E-06 | 0.00062 | 3.74E-10 |
| Thai1 | DCH011 | 0.38208 | 0.58009 | 0.08175 | 0.35292 | 0.29366 |  | 0.94102 | 5.15E-08 | 0.00051 | 9.09E-12 |
| Thai1 | DCH012 | 0.57067 | 0.91993 | 0.58606 | 0.31378 | 0.94102 | 0.23852 |  | 3.20E-05 | 0.00324 | 2.51E-09 |
| Thai2 | DCH007 | 0.00362 | 9.48E-08 | 1.65E-09 | 4.08E-10 | 1.83E-06 | 5.15E-08 | 3.20E-05 |  | 0.91768 | 0.47404 |
| Thai2 | DCH009 | 0.13881 | 0.00021 | 2.41E-06 | 6.31E-07 | 0.00062 | 0.00051 | 0.00324 | 0.91768 |  | 0.27235 |
| Thai3 | CHI034 | 1.38E-06 | 6.68E-13 | 3.78E-16 | 1.57E-17 | 3.74E-10 | 9.09E-12 | 2.51E-09 | 0.47404 | 0.27235 |  |

- L. 217: Please replace “form” by “from”

We have corrected it.

- L. 224: Please avoid ambiguous sentences such as “when we mapped the Thai1 population on the Coorghi skeleton”

We have corrected it.

- L. 234: Please replace “markup” by “makeup”

We have corrected it.
