# Supplementary material for: Indian genetic heritage in Southeast Asian populations
Source: PLoS Genet. 2022 Feb 17;18(2):e1010036. doi: 10.1371/journal.pgen.1010036 (PMC8853555; doi:10.1371/journal.pgen.1010036)
Supplement: Attachment — Submitted filename: response_to_reviewers_revision2.docx [file pgen.1010036.s020.docx]

**Editor’s comments**

The revised manuscript was fully evaluated at the editorial level and by one of the previous peer reviewers. As you will see, reviewer #2 still has some major concerns regarding the work. We agree with those concerns; in particular, we note that in its current form, the manuscript is still difficult to follow A more precise distinction of the population samples (both newly studied and collected from the literature) that were used as cases to test the hypotheses stated and the ones that were uses as reference populations, and a detailed description (in terms of geographic location and linguistic affiliation) of the whole dataset should be given in the beginning of results.; and maybe also in beginning of the results’ sections, where the hypotheses being tested should also be described.

In this revised manuscript, we highlighted populations which were newly genotyped in this study by adding an asterisk after population names in all the figures. In the case of PCA and ADMIXTURE analyses and biplot of *f_3_*-statistics, all population names were listed in the figures. A list of all populations used as target groups for the qpWave, qpAdm, qpGraph, and haplotype-based analyses appears in Table 1. For qpWave and qpAdm analyses, lists of all the target populations and surrogates we used are available in Supplementary Table 2. We have also mentioned all East and Southeast Asian (ESEA) surrogates, the surrogate for the deeply diverged East Eurasian hunter-gatherer lineage, and all qpWave/qpAdm outgroups in the text. For qpGraph analysis, all reference populations are shown in the figures (Fig. 6, S2-4 Fig.) and are listed in the Materials and Methods section. We added a list of populations used for the haplotype-based analysis in Supplementary Table 1. We also added information about geographic location and linguistic affiliation of all populations in the dataset in Supplementary Table 1.

There is also the lack of consistency in the naming of the populations throughout the paper, as mentioned by reviewer 2 – please be consistent on the naming of the population samples. Finally, the discussion section reads more as a summary of the results rather than a discussion.

Furthermore, we are concerned about overlap with the recent work from Kutanan et al. in MBE. Because that publication appeared during the revision of the current submission, we are willing to consider a revised manuscript for publication. However, for a revised manuscript to be successful, it will need to not only address concerns regarding reasoning and presentation, but also to explicitly acknowledge and comment on the work from Kutanan et al., noting which aspects of the current submission confirm, complement, and potentially extend the MBE paper.

We disagree that there is a major overlap between our work and that by Kutanan et al. in MBE. On the contrary, since Wibhu Kutanan is our co-author too, we tried to avoid an overlap in populations studied and in research questions at the research planning stage. In this revised manuscript, we tried to emphasize novel genotyping results and novel analytical conclusions wherever it was relevant, and we added a paragraph in the Discussion devoted to comparing our results to those by Kutanan et al. We replied to comments by Reviewer 2 and editorial comments point by point below.

Additional editorial comments:

•	Intro, Lines 89-93 – this is no longer true given Kutanan et al.

Here is a quote of lines 89-93 (from the previous manuscript version):

“Some genome-wide studies previously documented South Asian admixture in a few MSEA populations [11-13], but most ethnic groups in the region remain unexplored, especially Austroasiatic-speaking populations from Thailand which have strong Indian cultural connections. Some previous studies of MSEA populations did not focus on South Asian influence in the region, and South Asian ancestry was usually overlooked in those studies [14-16].”

We disagree with this comment. We do not find anything untrue in these sentences after Kutanan et al., 2021 was published. Below we clarify our statement phrase by phrase.

Quote

“Some genome-wide studies previously documented South Asian admixture in a few MSEA populations [11-13]”

In this phrase we acknowledged previous studies which found South Asian admixture in MSEA populations, including Kutanan et al., 2021 (reference 13).

Quote

“but most ethnic groups in the region remain unexplored, especially Austroasiatic-speaking populations from Thailand which have strong Indian cultural connections”

MSEA consists of Myanmar, Thailand, Malaysia, Laos, Cambodia, and Vietnam. Eberhard et al, 2020 reported numbers of indigenous languages spoken in these countries as following; Myanmar, 114 languages; Thailand, 51 languages; Malaysia, 112; Laos, 73; Cambodia, 20, and Vietnam, 93. We do not think that 33 ethno-linguistic groups from Thailand and Laos newly genotyped in Kutanan et al., 2021 would falsify the statement that most populations in the region (MSEA) remain unexplored. Nevertheless, in the current manuscript version we toned the phrase down by changing the word “most” to “many”.

In our study, we selected Austroasiatic-speaking populations which are highly influenced by Indian culture or populations which are likely to have connections with ancient Indianized states (i.e., populations that inherited languages and culture from those states). In Kutanan et al., 2021, there was only one population, Mon, which fits these criteria. And Mon is the only Austroasiatic-speaking population which was reported to harbor South Asian admixture by Kutanan et al., 2021. In the present study, we reported four Austroasiatic-speaking populations from Thailand with South Asian admixture (all of them are newly genotyped populations, Khmer, Kuy, Mon, and Nyahkur), which is more than any studies ever reported. Thus, the fact that not many Austroasiatic-speaking populations from Thailand which have strong Indian cultural connections were explored before our current study is correct. We note that the Mon groups studied by Kutanan et al., 2021 and in the present study come from different locations in Thailand.

Quote

“Some previous studies of MSEA populations did not focus on South Asian influence in the region, and South Asian ancestry was usually overlooked in those studies [14-16]”

We acknowledged previous studies which demonstrated South Asian ancestry in MSEA populations in the first sentence. In this sentence we mention studies which investigated MSEA populations but did not focus on South Asian ancestry in MSEA populations. These studies are the following: HUGO Pan-Asian SNP Consortium, 2009; Wangkumhang et al., 2013; and Liu et al., 2020. This statement is also correct. Even so, we rephrased the sentence by changing “usually” to “sometimes”.

- Intro, lines 96 – this sentence sounds weird – please rephrase

Quote of line 96 from the previous version: “In Thailand 51 indigenous languages from five major language families are attested [1].”

We rephrased to “In Thailand, 51 indigenous languages from five major language families are spoken [1]” (lines 94-95)

- Intro, lines 111-113 – “Khmer from Thailand is a Northern Khmer speaking population, which is closely related to Khmer…” is also strange -> please rephrase to Cambodia Khmer ; also it is not really clear what you intended to say with Khmer being descendants of people from ancient Khmer states, since this would be what you expected; or is it that you want to say that the Khmer comprise an ancient group/ present in Thailand for a significant number of years (whose number should be described in the text)? There are other sentences like this when referring to other population groups that I ask the authors to rephrase.

We have rephrased to Cambodian Khmer as suggested. Concerning the phrase “Present-day Khmer are likely to be descendants of people from ancient Khmer states”. We meant that the present-day Khmer in Thailand is a population which inherited language and culture form ancient Khmer states. Furthermore, present-day Khmer in Thailand also live in the area that once was under the influence of ancient Khmer states. So, they are possibly descendants of people from the ancient Khmer states. We added the phrases below in the introduction to clarify this point.

“Various present-day populations are possibly descendants of people from ancient Indianized states in the region, as they have inherited languages and culture from those states, and they reside in regions that once were within the territory of the ancient states [6].” (lines 92-94)

•	Results, lines 146-147 – change “is maximized” to “is enriched in”

We have rephrased as suggested (line 148).

•	Results, line 153 – I think “biplot” instead of “scatterplot” is more specific

We agree. We have rephrased as suggested (line 153).

•	Results, lines 180-182 – Sentences that start with a number, or that contain a number between 1-10, the numbers should be written down.

We have corrected them (line 180).

•	Discussion, lines 324-325 – this is no longer true given Kutanan et al.

Quote of lines 324-325 from the previous manuscript version:

“Previous studies reported South Asian admixture in a few populations from Southeast Asia [11-13]. Some studies analyzed the same or similar populations as those in the current study but did not focus on South Asian admixture [16, 36, 41].”

We disagree with this comment. These sentences are still correct. We break down sentence by sentence below.

Quote

“Previous studies reported South Asian admixture in a few populations from Southeast Asia [11-13].”

We cited previous studies which found South Asian admixture in MSEA populations, including Kutanan et al., 2021 (reference 13). Nothing is incorrect about this sentence.

Quote

“Some studies analyzed the same or similar populations as those in the current study but did not focus on South Asian admixture [16, 36, 41].”

In this sentence, we cited Liu et al., 2020, Lazaridis et al., 2014, and Kutanan et al., 2014. We used data from Liu et al., 2020 (Cham, Ede, and Giarai) and Lazaridis et al., 2014 (Cambodian Khmer). In the current study, we newly genotyped many individuals (Khmer from Thailand, Kuy, Mon, and Nyahkur) previously studied by Kutanan et al., 2014 from the perspective of uniparental markers only. Overall, we analyzed exactly the same individuals as in those studies. Those studies did not focus on South Asian/Indian admixture in MSEA populations and did not report that admixture, and we found South Asian admixture in some populations from those studies. We do not understand in what way the study by Kutanan et al. 2021 makes this statement incorrect.

•	Discussion, lines 517-518 – this is cryptic: what subtle differences or what similarities? How are these explained in terms of what is known about the history of the region?

We agree that these phrases sound ambiguous. We have removed the phrases.

•	Table S1 – Could you please include the country and language-family of the populations used?

We added the information to the Supplementary table as suggested.

•	Figure 5 – should read “Sgaw Karen”

We have corrected it.

Other comments regarding Analyses:

•	As noted by reviewer 2, there is a lack of evidence to support the splitting of the Thai groups.

We have removed all Thai groups from the biplot of *f_3_*-statistics, from the qpWave, qpAdm, qpGraph, and haplotype-based analyses. We think that removing Thai from these analyses will not affect the novelty of our study as Kutanan et al, 2021 recently reported South Asian admixture in the same Thai population (published data from Lazaridis at al., 2014). We just kept Thai in the PCA and ADMIXTURE analyses where individuals are treated independently.

•	Please use the f3-statististics, qpAdmix and qpGraph to demonstrate new results that complement Kutanan et al.

A great majority of our results remains novel even after the publication by Kutanan et al. 2021. In this study, we aimed to explore South Asian (Indian) ancestry in Austroasiatic-speaking populations from Thailand, which are heavily influenced by Indian culture, and populations from other MSEA countries from published data, which potentially harbor South Asian ancestry. We found South Asian ancestry in eight MSEA populations for whom it has not been reported before (four newly genotyped, four from published data). We discovered South Asian admixture in populations from Cambodia and Vietnam for the first time. We also for the first time used qpGraph to directly test Austronesian-related ancestry in various Kra-Tai-speaking populations, with our results supporting genetic connections between populations of the two language families, as suggested before by a linguistic hypothesis. Throughout the Results, Discussion and figures we tried to emphasize what results are novel and what results are confirmatory. Here are some examples with quotes from the main text. We cited Kutanan et al., 2021 as reference 13.

Confirmatory results (quotes from the latest manuscript version):

“The genetic interaction between Karen and Lawa was also observed by Kutanan et al. [13].” (lines 345-346)

“Akha, Lawa, Karen, Hmong, and Htin were officially recognized as hill tribes by the Hill Tribe Development and Welfare Programme of the Department of Public Welfare in Thailand [21]. Maniq and Mlabri are the last two hunter-gatherer groups in Thailand [45]. We failed to detect South Asian ancestry in these seven populations. The result is consistent with a recent study by Kutanan et al. [13], which investigated eight hill tribes from Thailand and detected no South Asian admixture.” (lines 366-370)

“A Malay group from Singapore was modeled by qpGraph as a 3-way admixture involving sister groups of Atayal, Mlabri, and South Asian populations (S4H Fig, S3 Table). Malay is an Austronesian language. It is not surprising that the Malay harbor some ancestry from a source related to Atayal, an Austronesian-speaking population from Taiwan. A previous study found admixture from an Austroasiatic-speaking population in Austronesian populations from Indonesia [5]. We also detected the same signal in Malay, which is represented by ancestry from a sister group of Mlabri (S4H Fig, S3 Table). SOURCEFIND also reveals the presence of South Asian admixture in Malay (Fig 7). An earlier work by Kutanan et al. [13] also reported a similar genetic makeup of a Malay-speaking group from Thailand.” (lines 375-383)

“The inferred South Asian admixture date in Mon is around 600 YPB (Fig 8), which fits the Mon Independent Ramanya Polity period (1300 - 1539) in present-day lower Myanmar [22] and is close to a previous estimate by Kutanan et al. [13].” (lines 432-434)

“In concordance with an earlier study [13], we failed to detect South Asian admixture in relatively isolated "hill tribes" and in present-day hunter-gatherer groups from Thailand.” (lines 493-495)

Novel results or results extending those by Kutanan et al.:

“Our study revealed substantial South Asian admixture in various populations across Southeast Asia (~2-16% as inferred by qpAdm). For four ethnolinguistic groups genotyped in our study (Khmer from Thailand, Kuy, Mon, and Nyahkur) and four groups from published data (Cambodian Khmer, Cham, Ede, and Giarai [16, 40]) South Asian admixture was not reported before in the literature, and we report it here. Thus, we for the first time demonstrate South Asian admixture in populations from Cambodia and Vietnam, extend earlier results detecting this ancestry component in Thailand [13], and confirm detection of this ancestry component in Myanmar and Singapore [12]. A recent study [13] also observed South Asian ancestry in various groups of Thai, a Malay group from Thailand, and Mon from different locations in Thailand other than in the current study.” (lines 479-487)

“Prior to our study, Mon was the only known Austroasiatic-speaking population in Mainland Southeast Asia with confirmed South Asian admixture [13]. We also detected South Asian ancestry in Mon (from different location other than in Kutanan et al., 2021 [13]) and four other Austroasiatic-speaking populations in Thailand and Cambodia (three newly genotyped populations and one from published data) which inherited languages and culture from ancient Indian-influenced states. The South Asian admixture dates inferred in our study fit the historical context of ancient Indian-influenced states.” (lines 487-493)

“In the current study, we analyzed populations from six branches of the Austroasiatic language family: 1) Khmuic (Htin and Mlabri), 2) Katuic (Kuy), 3) Palaungic (Lawa), 4) Monic (Mon and Nyahkur), 5) Khmeric (Khmer from Thailand and Cambodia), and 6) Aslian (Maniq) [1]. Kutanan et al. 2021 [13] investigated populations from four branches of the Austroasiatic language family in Thailand and revealed three genetic clusters, which fitted rather well with linguistic branches: 1) Khmuic and Katuic, 2) Paluangic, and 3) Monic. However, genetic profiles of some Austroasiatic-speaking populations in our analysis do not fit well with linguistic branches as they resemble populations from different branches, and there is genetic heterogeneity within linguistic branches. We also observed genetic differences between the Khmuic, Paluangic, and Monic groups in our study, i.e., we detected additional Tibetan-related ancestry in a Paluangic population (Lawa) as compared to Khmuic groups (Htin and Mlabri), while South Asian admixture in Monic populations (Mon and Nyahkur) distinguished them from the other two groups. Even though Khmuic and Katuic-speaking populations in Kutanan et al. 2021 [13] demonstrated a similar genetic makeup, genetic profiles the of Khmuic and Katuic-speaking groups in our study are distinct as a Katuic group (Kuy) harbors South Asian admixture, while that ancestry is missing in Katuic groups from Kutanan et al., 2021 [13] (Bru and Soa) and in Khmuic groups from both studies. Kuy’s genetic profile is similar to that of Khmer from Thailand and Nyahkur, Austroasiatic-speaking populations from the Khmeric and Monic branches, respectively. We also observed genetic heterogeneity within linguistic branches of the Austroasiatic family, reflecting different interaction with other populations. Tibetan-related ancestry is present in Mon, but the ancestry is absent in Nyahkur, another Monic-speaking group in our study. Khmer from Cambodia harbor Atayal-related admixture, but that ancestry is lacking in Khmer from Thailand. Maniq, an Aslian-speaking population, is distinct from other Austroasiatic-speaking populations, as their predominant genetic component is from an Onge-related deeply diverged East Eurasian lineage.” (lines 500-522)

“Some previous studies suggested a genetic link specifically between Austronesian-speaking and Kra-Dai- speaking populations, relying on f3- or f4-statistics [13, 51, 52]. We for the first time used qpGraph to directly estimate Atayal-related ancestry in dozens of Kra-Dai-speaking populations from China and Vietnam (~3-38% as inferred by qpGraph). Our results strengthen the previous results that Austronesian and Kra-Dai-speaking populations share a component of ancestry unique to those two groups.” (lines 524-529)

Reviewer's Responses to Questions

Comments to the Authors:

Please note here if the review is uploaded as an attachment.

Reviewer #2: I appreciate the authors making substantial revisions based on previous comments. While I think in the current version the authors at times still deviated from their main thesis of South Asian admixture in MSEA populations, the manuscript reads much better now.

My only major comment is still the practice of splitting of Thai into 3 groups. While the authors clarified that they decided to split Thai into 3 groups based on multiple analyses (PCA, ADMIXTURE, qpGraph, etc.), and I don’t deny that the seven Thai1 individuals may have a different admixture profile as the single Thai3 individual, the practice still appears arbitrary and unmerited. Given an admixed population, there are naturally variation in the admixture profile. That does not mean individuals at two ends of the spectrum are representatives of two populations. Imagine Latino individuals living in the United States. Sampling of Latino individuals from different parts of U.S., or even from the same city in U.S., will appear to have different proportion of ancestry. You might easily find some who carry African ancestry while others do not. That fact by itself does not mean there are multiple sub-populations within Latinos. By the authors’ own implicit admission, they have no other information on the Thai individuals (lines 455-456). Thus, there is no support from other sources (language, culture, self-identity, etc.) that would suggest it is appropriate to divide the Thai group into three. This is also based on very limited sampling (Thai2 and Thai3 have 2 and 1 individual, respectively). Such a contrived categorization ought to require a much larger study of the fine-scale structure of Thai, not based on a total of 10 individuals; taken to the extreme, this practice could be considered racist (discretizing a sample of individual based on their genetic profile). And ultimately, I see no additional scientific insight brought about by splitting Thai into three subgroups, other than the fact that “you can”.

We have removed all Thai groups from the biplot of *f_3_*-statistics and from qpWave, qpAdm, qpGraph, and haplotype-based analyses. We think that removing Thai from these analyses will not affect our study as Kutanan et al, 2021 recently reported South Asian admixture in the same Thai population (published data from Lazaridis at al., 2014.). We just kept Thai for PCA and ADMIXTURE analyses, where individuals are treated separately.

Other comments:

1. The author should be more consistent when referring to populations in the manuscript. For example, multiple times they refer to “Indian” population (Line 147), but the Figure says “SAS”.

SAS is our meta-population label for all South Asian populations. In the context quoted by the reviewer, we wanted to emphasize that the component was enriched in Indian populations, not other South Asian populations from Pakistan or Bangladesh. However, we agree that the phrase we used in the previous manuscript was ambiguous. We rephrased it to clarify the text:

“At 12 hypothetical ancestral populations, Mon, Khmer from Thailand, Kuy, Nyahkur, Burmese, Thai, Cambodian Khmer, Cham, Ede, Giarai, and Malay (data for four former populations were generated in this study) demonstrated a “light pink” ancestry component (accounting for more than 5% of their ancestry, on average) that is enriched in South Asian populations such as Irula and Mala from Southern India (Fig 3). (lines 144-149)

There are also times they refer to Onge in the text, but NEG in the corresponding figure. In this case Onge and ESEA or South Asians are different levels of population labels.

We have changed the meta-population label of Onge to NEGA (Andamanese Negritos) and tried to use consistent levels of labels in the text: either population level for all, or meta-population level for all.

2. The authors insisted on showing only the skeleton graph in Figure 6. I would prefer that the skeleton graph be moved to Supplemental Figure and instead show one or two graphs illustrating the main results instead. At the very least, a graph for each population examined in Figure 5 should be made in the supplement. Table S3 is very difficult to decipher and would help a lot with a graphical representation.

We agree with this comment, and we moved the skeleton graphs to a Supplementary Figure (S2 Fig), as suggested. We plotted one representative best-fitting graph for each MSEA target population (all populations mentioned in Fig. 5 and Table 1) in Fig. 6 and S4 Fig. All the other models are still listed in Table S3. We hope that now our analytical procedure and results are much clearer.

Other minor comments:

Line 43-44: this sentence should be tuned down a bit. There is evidence of gene flow, which “may have been” responsible for the spread of Indian culture in the region, but there’s no direct demonstration in this paper.

We rephrased to sentence to “The results suggest that migration of Indian populations to MSEA may have been responsible for with the spread of Indian culture in the region.” (lines 43-44)

Line 71: reword “nearly substantial”

We rephrased to “Andamanese hunter-gatherers (Onge and Jarawa) and MSEA Negritos are present-day populations with substantial proportions of ancestry from the deeply diverged East Eurasian hunter-gatherer lineage [4, 5].” (lines 67-69)

Line 134: should it be Eastern Southeast Asians for ESEA?

ESEA in our manuscript stands for East and Southeast Asians.

Line 146: it would be helpful if you can name the color of the ancestry component you’re referring to.

We added the color name (light pink) in the text. “At 12 hypothetical ancestral populations, Mon, Khmer, Kuy, Nyahkur, Burmese, Thai, Cambodian, Cham, Ede, Giarai, and Malay (the first four populations are newly genotyped data from this study) demonstrated a “light pink” ancestry component (accounting for more than 5% of their ancestry, on average) that is enriched in South Asian populations such as Irula and Mala from Southern India (Fig 3).” (lines 144-149)

Have all data underlying the figures and results presented in the manuscript been provided?

Large-scale datasets should be made available via a public repository as described in the PLOS Genetics data availability policy, and numerical data that underlies graphs or summary statistics should be provided in spreadsheet form as supporting information.

Reviewer #2: No: Authors indicated data is on Reich's Lab website. I don't see them.

We had a statement in the previous version of the manuscript that the data will be available after acceptance of the manuscript. We stated in the “Additional data availability information” section: “Tick here if the URLs/accession numbers/DOIs will be available only after acceptance of the manuscript for publication so that we can ensure their inclusion before publication.”
